# Comprehensive transcriptional analysis of ethylene and softening regulation in plums with distinct climacteric ripening behaviors

**DOI:** 10.1186/s12870-025-06932-w

**Published:** 2025-07-12

**Authors:** Po-Kai Huang, George A. Manganaris, Ksenija Gasic, Christopher A. Saski, Diane M. Beckles, Carlos H. Crisosto

**Affiliations:** 1 Department of Plant Sciences, University of California, Davis, 95616 USA; 2 Department of Agricultural Sciences, Biotechnology & Food Science, Cyprus University of Technology, Lemesos, 3603 Cyprus; 3 Department of Plant and Environmental Sciences, Clemson University, Clemson, SC USA

**Keywords:** *Prunus salicina*, Transcriptional regulation, Ethylene, Fruit softening, Climacteric, Suppressed climacteric, Non-climacteric

## Abstract

Understanding the molecular mechanisms underlying variations across climacteric categories in tree crops remains challenging, particularly due to the limitations of applying conventional genetic approaches. In this study, we examined genetically related Japanese plum (*Prunus salicina* Lindl.) cultivars with distinct ethylene production and responses during ripening: ‘Santa Rosa’ (climacteric fruit), ‘Casselman’ (suppressed-climacteric fruit), and ‘Sweet Miriam’ (non-climacteric fruit). Our primary objective was to comprehensively investigate transcriptional differences related to ethylene and fruit softening across three on-tree developmental stages: “Green” (early development), “Mature” (pre-climacteric or early climacteric), and “Ripe” (climacteric). By integrating information from Phytozome and Dicot PLAZA databases, we identified complete gene families for six ethylene-related and seven softening-related genes. Multi-developmental stage RNA-seq clustering revealed that “Late” genes, which increase in expression in ripe fruit, are associated with physiological differences among climacteric categories. We demonstrated that ‘Casselman’ aligns more closely with ‘Sweet Miriam’ than ‘Santa Rosa’ at the transcriptional level for these “Late” genes, consistent with their shared low ethylene production. Gene expression analyses revealed additional factors beyond ethylene, including jasmonate-related genes and *NAC* transcription factors as influencing climacteric ripening behavior. To extend our findings beyond the three representative cultivars, we performed qPCR on additional cultivars harvested under different field conditions and years, including ‘Friar’ and ‘Fortune’ as climacteric plums and ‘Late Santa Rosa’ and ‘Angeleno’ as suppressed-climacteric plums. *PpACO1*, *PpPL1*, *PpBGAL16*, *PpNAC2*, and *PpJID1* expression patterns were conserved across cultivars and experimental conditions. Our findings provide novel insights into the transcriptional regulation of climacteric ripening and offer a strategic framework for future genetic studies in plum and other tree crops.

**Supplementary Information:**

The online version contains supplementary material available at 10.1186/s12870-025-06932-w.

## Background

Fruit ripening is a complex physiological process categorized based on patterns of respiration, ethylene production, and ethylene response. During ripening, climacteric fruits exhibit a burst of respiration and ethylene production, with the latter being autocatalytic upon ethylene exposure. In contrast, non-climacteric fruits lack these characteristics [1, 2]. Suppressed-climacteric fruits display intermediate patterns: carbon dioxide and ethylene production are low, but increases like climacteric fruit when exposed to exogenous ethylene [3–5]. Since ethylene influences fruit storage and quality [6], cultivars with varying climacteric ripening behaviors offer valuable opportunities for understanding mechanisms to improve consumer satisfaction and reduce postharvest losses. In this study, we used the Japanese plum (*Prunus salicina* Lindl.) as a model system to investigate the climacteric regulation of softening, an important trait for fruit quality and postharvest handling.

In plants, 1-aminocyclopropane-1-carboxylate (ACC) synthase (*AC*S) and ACC oxidase (*ACO*) are the two key genes involved in ethylene biosynthesis [2, 7, 8]. This pathway is transcriptionally regulated, and two ethylene production systems are defined based on feedback mechanisms: System 1, found in young fruit, exhibits autoinhibition, while System 2, active in ripening fruit, is autocatalytic [9]. The primary components of the ethylene signal transduction pathway in tomato are well-defined, including receptors ETHYLENE RESPONSE (*ETR*s) and CONSTITUTIVE TRIPLE RESPONSE (*CTR*s), as well as regulators ETHYLENE INSENSITIVE2 (*EIN2*), ETHYLENE INSENSITIVE3 (*EIN3*), EIN3-like (*EIL*s), and ETHYLENE RESPONSE FACTORS (*ERF*s) [2, 7, 8]. While some of these ethylene-related genes have been studied in stone fruits, most studies only examined a subset of the gene family.

Fruit softening is an irreversible, ethylene-regulated process in climacteric fruits. It mainly involves changes to the primary cell wall, including pectin modification and cell wall loosening [10, 11]. In stone fruits, pectin metabolism is most studied in peach, where pectinases, including endo-polygalacturonases (*PG*) and pectate lyases (*PL*), along with pectin methylesterases (*PME*) and β-galactosidases (*BGAL*) are functionally and transcriptionally linked to softening [12–18]. Cell wall loosening, on the other hand, is mediated by expansins, endo-1,4-β-glucanases (*EG*), and xyloglucan endotransglucosylase/hydrolases (*XTH*). Although some studies have documented changes associated with cell wall loosening during peach ripening [19–22], comprehensive analyses of the underlying gene regulation remain lacking in stone fruits.

Understanding the mechanisms underlying different climacteric ripening patterns remains challenging. Comparative studies of climacteric and non-climacteric species, such as tomato and strawberry [1, 23, 24], provide limited insights due to their large phylogenetic distance. Generating genotypes with different climacteric patterns within the same species for QTL mapping, as in melons [25], addresses this issue but is not feasible for tree species due to their long generation times. Japanese plum provides an attractive model for studying climacteric ripening in tree crops, as cultivars derived from somatic “bud sport” mutations of ‘Santa Rosa’ share closely related genetic backgrounds but exhibit diverse climacteric ripening behaviors [5, 26]. ‘Santa Rosa’ (SR) is climacteric, while ‘Sweet Miriam’ (SM) is non-climacteric. ‘Casselman’ (CM) and ‘Late Santa Rosa’ (LSR) are considered suppressed-climacteric plums, exhibiting delayed ripening without external ethylene. Distantly related cultivars also show variation in ripening behavior, with ‘Fortune’ (FT) and ‘Angeleno’ (Ang) being climacteric and suppressed-climacteric, respectively [27, 28].

Several biological differences have been associated with the distinct climacteric ripening behaviors of SR and SM [29–32]. However, the softening differences among climacteric categories require further investigation. In a comprehensive report, Minas et al. [5] demonstrated that the softening rate in response to external ethylene exposure was associated with climacteric categories, varying from low (non-climacteric) to low-to-intermediate (suppressed-climacteric) and high (climacteric). However, the transcriptional mechanisms and specific genes underlying these differences remain unclear. Studying softening-related genes in plum is challenging, as the reference genome is not fully annotated and many softening enzymes belong to diverse gene families, many of which remain functionally uncharacterized [13, 15, 17, 18]. *De novo* prediction of these enzymes is time-intensive, and more efficient strategies are required to identify all genes across gene families.

Previous genetic studies did not include suppressed-climacteric cultivars, leaving open the question of whether the differences between climacteric and non-climacteric responses also apply to suppressed-climacteric cultivars. Furthermore, using only one cultivar to represent each climacteric category raises the question of whether these findings are generalizable to other cultivars. Here, we address these knowledge gaps by including suppressed-climacteric CM alongside SR and SM. We examined three developmental stages: “Green” (early development), “Mature” (pre-climacteric or early climacteric), and “Ripe” (climacteric), to categorize genes as “Early” or “Late” based on their expression dynamics. We integrated information from two genome databases to comprehensively identify all relevant genes. These data were used to investigate: (1) the genes related to softening differences across different climacteric ripening patterns in plums, (2) whether the expression patterns of these genes in suppressed-climacteric plums are more similar to non-climacteric plums than to climacteric plums, and (3) whether the findings can be extrapolated from these three to other plum cultivars.

## Materials and methods

### Plant material

For RNA-seq analysis, fruits from Japanese plum (*Prunus salicina L.*) cultivars ‘Santa Rosa’, ‘Casselman’, and ‘Sweet Miriam’ were harvested from Reedley, CA, in 2017 at three developmental stages: “Green”, “Mature”, and “Ripe”. The developmental stages were defined as follows: “Green” (pit hardening stage, S2), “Mature” (pre-climacteric stage, S3/S4, firm fruit with green-yellow flesh right under the skin), and “Ripe” (climacteric stage, S4-II, softening texture with yellow-red flesh right under the skin), according to the California Tree Fruit Agreement [33]. Five fruit per tree served as a biological replicate, with a total of three replicates per cultivar-by-developmental stage. Fruits were packed in cardboard boxes and transported to the UC Kearney Agricultural Research and Extension Center (KARE) for processing the same day. The frozen tissues were then shipped to Clemson University for RNA extraction and RNA-seq experiments.

For qPCR analysis conducted in 2024, fruits from cultivars ‘Friar’, ‘Fortune’, ‘Casselman’, ‘Angeleno’, and ‘Sweet Miriam’ were harvested from Reedley, CA, while ‘Santa Rosa’ was obtained from a local farmer in Brentwood, CA, and ‘Late Santa Rosa’ was donated by farmers in Vacaville and Sebastopol, CA. Fruits were collected from five trees (biological replicates) per cultivar, with eight fruits per tree, except for ‘Late Santa Rosa’, which was collected from two trees. All fruits were harvested at the “Ripe” stage, and except for ‘Santa Rosa’, cultivars were stored at room temperature for 2–3 days to ensure uniform ripening before processing.

Except for the fruits donated by local farmers, all plum samples were collected from commercially managed orchards or from the experimental orchard at KARE, both following commercial practice guidelines recommended by the University of California Cooperative Extension and implemented by KARE staff [34]. Trees grafted onto Citation rootstock were planted in a traditional open vase system at a spacing of 12 feet × 18 feet (tree × row), with a planting density of approximately 202 trees per acre.

For both analyses, fruits were washed, peeled, and the flesh were frozen in liquid nitrogen and stored at −80 °C until further analyses. Harvest dates varied by cultivar and are provided in Table S1.

### RNA-seq library preparation and sequencing

RNA in flesh was extracted following the protocol of Meisel et al. [92]. RNA quality was assessed using Nanodrop 8000 spectrophotometer (ThermoFisher Scientific, USA) and Bioanalyzer 2100 (Agilent Technologies, USA), with all samples achieving an RNA integrity number of at least 7. RNA was quantified using Qubit fluorometer (ThermoFisher Scientific, USA). Libraries were prepared using NEBNext Ultra II RNA Library Prep Kit for Illumina (New England Biolabs, USA), pooled in equimolar ratios, and sequenced on an Illumina NovaSeq 6000 S4 flow cell, generating paired-end reads (2 × 150 bp). RNA-seq data are available via the NCBI Sequence Read Archive (SRA) (BioProject: PRJNA1249002).

### Comprehensive ethylene- and softening-related gene identification

Candidate genes were identified by integrating annotations from Phytozome v13 [35] and Dicots PLAZA 5.0 [36]. Enzyme groups were classified using Enzyme Commission (EC) annotations from Phytozome, retrieved via the `biomaRt` R package [37]. Gene families within enzyme groups were defined using homologous relationships identified through the “Integrative Orthology Viewer” in Dicots PLAZA, which combines multiple approaches to generate a consensus view of gene orthology. To ensure comprehensive coverage, we included all potential genes by taking the union of the gene lists from both databases. For target genes without an EC number, orthology-based identification was used. Detailed enzyme descriptions and EC numbers are provided in Table S2.

### Published RNA-seq data downloading

The RNA-seq FASTQ datasets were downloaded from the NCBI SRA using the SRA Toolkit. Only two datasets (PRJNA384370 [29] and PRJNA752255 [38]) relevant to SR-derived plums are available. We subset the data from Farcuh et al. (2017) (PRJNA384370) to focus on the SR and SM at the “Ripe” stage. The Salazar et al. (2022) study (PRJNA752255) provides data on SR fruit treated with gasified 0.14% 1-MCP for 20 h or dipped in 600 ppm Ethrel for 20 min. The raw sequencing data were processed and reanalyzed using our independently developed pipeline, with a focus on gene expression not reported in the original publications.

### RNA-seq data analysis

Sequencing reads were assessed for quality using FastQC [39] and then mapped to the peach reference genome (*Prunus persica* v2.1 from Phytozome v13) with the STAR aligner [40] using default settings. Count tables generated by STAR were merged and used as input for the voom method in `limma` package (v3.60.2) [41]. Model fitting was performed based on the guidelines in the limma-voom tutorial [42]. For visualization and comparison of target gene expression levels, values were presented on the Counts Per Million (CPM) scale.

Differential gene expression (DGE) analysis was performed with `limma` package using two criteria to identify candidate genes with sharp expression differences during late ripening in SR and distinct expression patterns across SR, CM, and SM. The first criterion filtered genes differentially expressed between the “Mature” and “Ripe” stages in SR, applying a 1.5-fold change threshold. The second criterion identified genes with significant developmental stage-by-cultivar interactions. Both criteria were based on a false discovery rate (FDR) threshold of 1%, using the Benjamini-Hochberg procedure [43].

GO and MapMan [44] annotations were obtained from Dicots PLAZA. GO enrichment analysis was performed using the `goseq` package (v1.56.0) [45], correcting for total count bias with an FDR cutoff of 0.05. MapMan enrichment analysis was conducted using `enricher` function in the `clusterProfiler` package (v.4.12.6) [46]. Weighted gene co-expression network analysis (WGCNA) was performed using the `WGCNA` package (v1.73) [47] with single-block, signed network, and power = 16. Hub genes were identified by Kleinberg’s score using `get_hub_kleinberg` function in the `GWENA` package (v1.14.0) [48].

### qPCR analysis

RNA was extracted from flesh using the protocol of Gambino et al. [93] with modifications and treated with DNase I (Zymo Research, USA) to remove genomic DNA. We used 2,000 ng of RNA for cDNA synthesis with the High-Capacity cDNA Reverse Transcription Kit (ThermoFisher Scientific, USA) with random primers. qPCR was performed on QuantStudio 3 using PowerUp SYBR Green Master Mix (ThermoFisher Scientific, USA). The expression of each target gene was normalized to *Initiation factor 5 A* (*IF5A*, Prupe.1G280900), as recommended by Kim et al. [94], and its stability was verified using our RNA-seq results. Primers were designed using NCBI Primer-BLAST [49] based on the peach reference genome and tested for sequence variants using the *Prunus salicina* Sanyueli Whole Genome v2.0 [50] from the Genome Database for Rosaceae (GDR) [51]. Primer sequences are listed in Table S3. *PpJID1-1* and *PpJID1-2* shared high sequence identity, and the primer sets could not distinguish between these homologs.

### Statistical analyses

All statistical analyses were conducted using the R language (R v.4.4.0). For pairwise comparisons, we first fitted a linear model using the `lm` function in the `stats` package and processed the results using the `emmeans` and `contrast` functions in the `emmeans` package [52]. Hierarchical clustering was conducted using the `hclust` and `cutree` functions in the `stats` package. Data visualization was performed using the `ggplot2` package [53].

## Results

### Comprehensive data integration identifies “early” and “late” ethylene biosynthesis genes in SR

To comprehensively identify all *ACS* and *ACO* genes, we integrated Enzyme Nomenclature (EC number) annotations from Phytozome with orthologous relationships from PLAZA Dicot. This approach revealed several *ACS* and *ACO* genes that have not been reported in previous peach or plum studies (Table 1).

**Table 1:** Ethylene biosynthesis genes in the Peach genome. Gene families are defined based on orthologous relationships from dicots PLAZA. Groups are categorized by expression patterns determined through hierarchical clustering, with “-” indicating expression undetected

Gene ID	Gene name	Family	Group	Described before (in peach/in plum)
Prupe.2G176900	*PpACS1*	ACS family1	1	Y/Y
Prupe.1G417800	*PpACS2*	ACS family1	-	Y/**N**
Prupe.2G283100	*PpACS3*	ACS family1	2	Y/**N**
Prupe.5G083500	*PpACS4*	ACS family1	2	Y/**N**
Prupe.5G106200	*PpACS5*	ACS family1	-	Y/**N**
Prupe.6G214400	*PpACS6*	ACS family1	-	Y/**N**
Prupe.7G213800	*PpACS7*	ACS family1	-	Y/**N**
Prupe.7G213900	*PpACS8*	ACS family1	1	Y/Y (ACS3 in Farcuh et al., 2019) [31]
Prupe.3G209900	*PpACO1*	ACO family1	1	Y/Y
Prupe.4G013800	*PpACO2*	ACO family1	3	Y/**N**
Prupe.7G212000	*PpACO3*	ACO family1	1	Y/Y
Prupe.1G490000	*PpACO4*	ACO family2	-	**N/N**
Prupe.2G251400	*PpACO5*	ACO family3	3	**N/N**
Prupe.3G060100	*PpACO6*	ACO family4	-	**N/N**
Prupe.5G064700	*PpACO7*	ACO family5	1	**N/N**
Prupe.6G230800	*PpACO8*	ACO family6	2	**N/N**
Prupe.7G221000	*PpACO9*	ACO family5	1	**N/N**

Genes not previously described in Peach or Plum are marked with a bold “N”

**Fig. 1:**
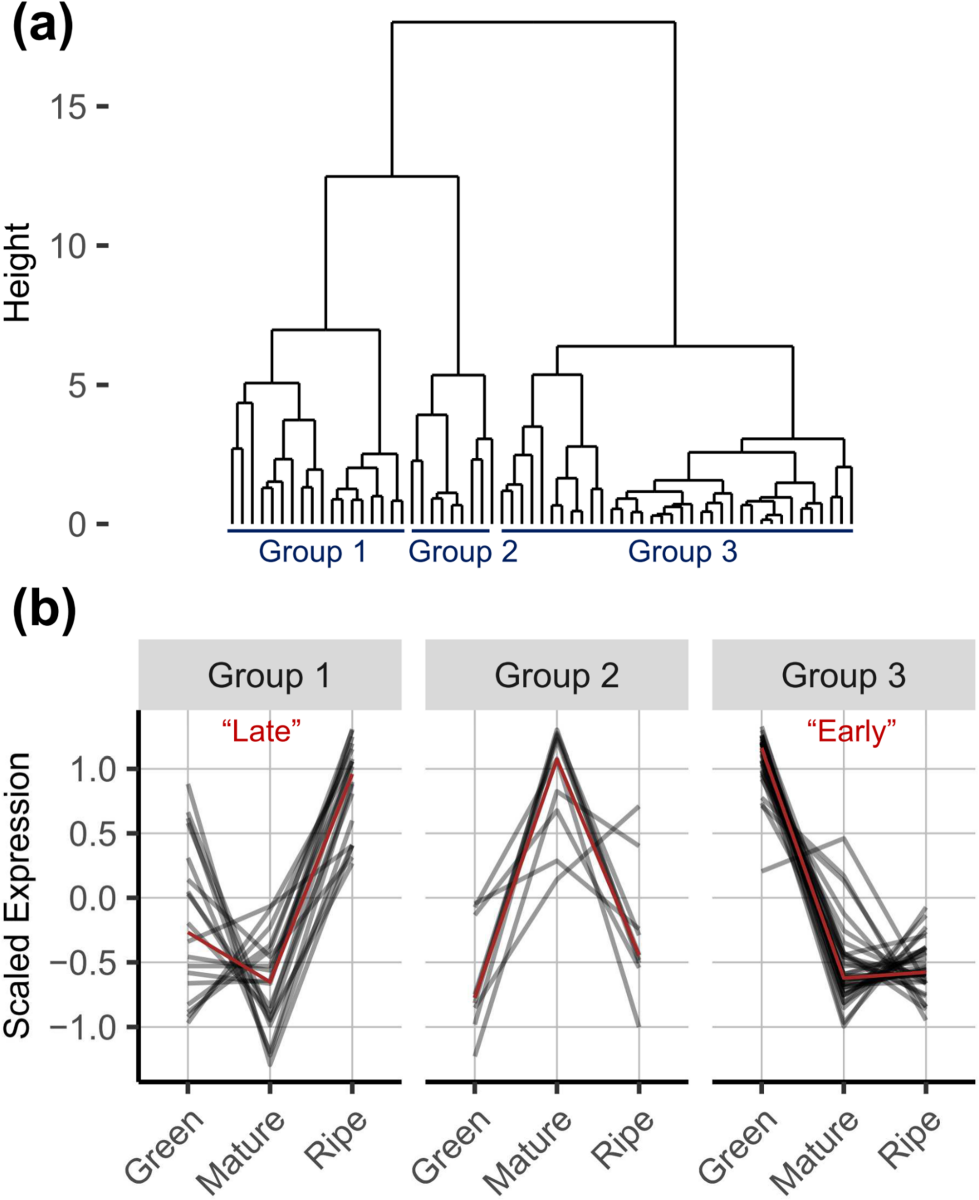
Ethylene-related genes are categorized by expression patterns across three developmental stages in ‘Santa Rosa’.** a** Hierarchical clustering reveals three groups, with Groups 1 and 3 corresponding to “Late” and “Early” genes, respectively. The dendrogram with gene names is provided in Fig. S1. **b** Standardized expression dynamics for each gene within the groups are shown, with the red curve indicating the median expression trend

Using the climacteric SR as a reference, we categorized all ethylene biosynthesis and signaling genes by their expression dynamics across “Green”, “Mature”, and “Ripe” stages through hierarchical clustering (Fig. 1). Only a subset of gene expression was detected, suggesting tissue or temporal specificity. Two distinct groups emerged: “Late” genes, which showed an increasing trend between the “Mature” and “Ripe” stages, corresponding to the transition from the pre- or early-climacteric to climacteric stage; and “Early” genes, which exhibited peak expression at the “Green” stage.

**Fig. 2:**
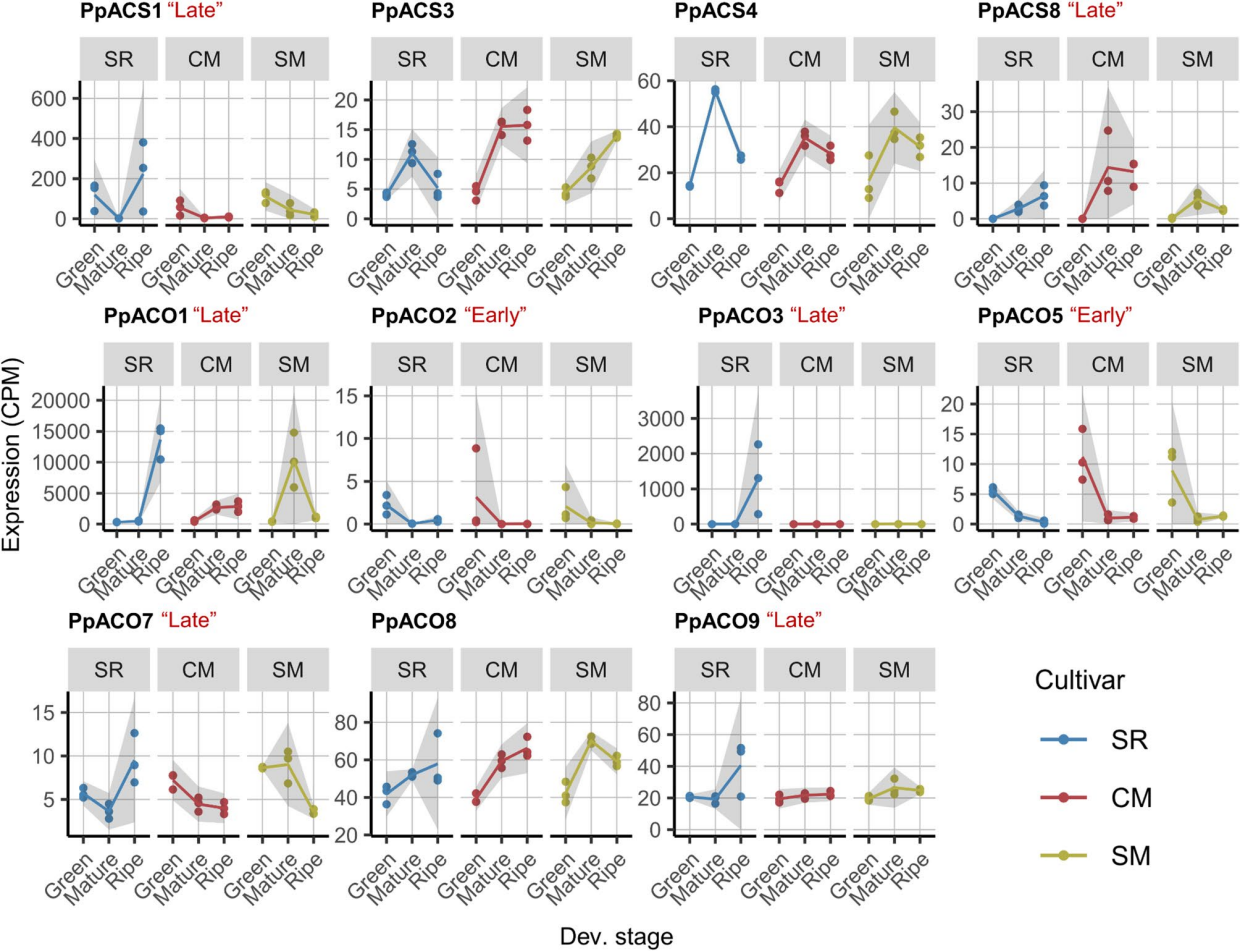
Ethylene biosynthesis genes, *ACS* and *ACO*, vary in expression patterns and levels. Each facet represents the expression dynamics in flesh across the “Green”, “Mature”, and “Ripe” stages for ‘Santa Rosa’ (SR), ‘Casselman’ (CM), and ‘Sweet Miriam’ (SM). The grey ribbon around each curve indicates the 95% confidence interval. Red labels (“Late” or “Early”) indicate the expression patterns

The expression of four out of the eight *ACS*, and seven out of the nine *ACO* were detected. Among them, *PpACS1*, *PpACS8*, *PpACO1*, *PpACO3*, *PpACO7*, and *PpACO9* followed the “Late” pattern, while *PpACO2* and *PpACO5* exhibited the “Early” pattern (Fig. 2). Even within the same expression category, variations were observed. For instance, *PpACS1* and *PpACO7* were moderately expressed at the “Green” stage, a trait not observed in other “Late” ethylene biosynthesis genes, suggesting developmental regulations. Expression levels also varied substantially within the same enzyme group: the highest expression of *ACO*, *PpACO1*, reached over 10,000 counts per million (CPM) at the “Ripe” stage, approximately 10-fold higher than *PpACO3*, the second-highest expressed gene.

In addition to the “Late” and “Early” patterns, *PpACS3* and *PpACS4* exhibited peak expression specifically at the “Mature” stage. Taken together, our workflow demonstrates the expression variation in ethylene biosynthesis genes, indicating distinct roles during different developmental stages. These differences would likely have been overlooked without a comprehensive analysis of all related genes.

### Not all “Early” and “Late” ethylene biosynthesis genes follow typical System 1 and 2 regulatory patterns in SR

Ethylene biosynthesis genes are classified as System 1 if highly expressed during early fruit development and independent of or negatively regulated by ethylene, whereas System 2 genes peak later and are positively regulated by ethylene. To determine whether the “Early” and “Late” genes correspond to System 1 and System 2 ethylene production, we examined their responses to ethylene using the published data from Salazar et al. (2022) [38]. In their study, SR fruits were harvested at the commercial maturity stage and treated with either 1-MCP, an ethylene inhibitor, or Ethrel, an ethylene precursor. We retrieved the raw sequencing data and reanalyzed them using our independently developed pipelines. Except for *PpACS1*, none of the genes below were examined in the original publication.

**Fig. 3:**
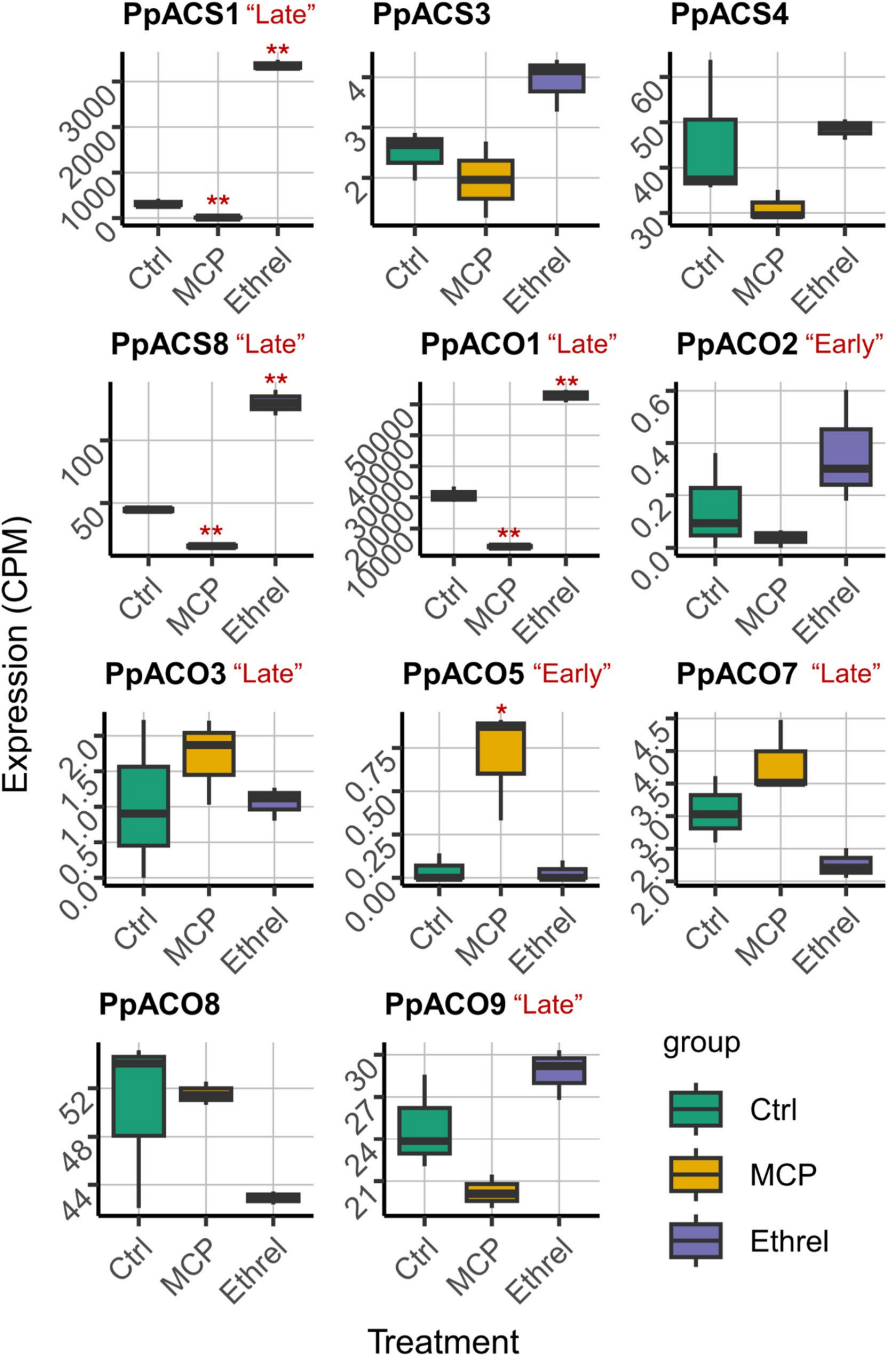
Responses of *ACS* and *ACO* to 1-MCP and Ethrel reveal that not all “Early” and “Late” genes in ‘Santa Rosa’ follow System 1 and 2 regulatory patterns. The raw sequencing data from Salazar et al. (2022) were reanalyzed using our independently developed pipeline, targeting a different set of genes than the original study. The fruit were treated with gasified 0.14% 1-MCP for 20 h or dipped in 600 ppm Ethrel for 20 min. Box colors represent treatments (Control = green, 1-MCP = yellow, Ethrel = purple). Boxes display the interquartile range, with horizontal lines indicating the median. Whiskers extend to the minimum and maximum values within 1.5 times the interquartile range. Asterisks indicate significance levels (*p < 0.05, **p < 0.01) based on Dunnett’s test comparing treatments to Control

As shown in Fig. 3, the expression of “Late” *ACS*, *PpACS1* and *PpACS8*, significantly decreased with 1-MCP treatment but was induced by Ethrel, exhibiting clear System 2 behavior. Interestingly, *PpACS3* and *PpACS4*, specifically expressed at the “Mature” stage, also showed similar responses, although the differences were not statistically significant.

The downstream ethylene synthesis gene, *ACO*, exhibited more complex responses (Fig. 3). Among the “Late” *ACO*, *PpACO1* and *PpACO9* displayed a System 2 pattern in response to 1-MCP and Ethrel. In contrast, *PpACO7* showed a reversed regulation, with expression levels decreasing after Ethrel treatment and increasing with 1-MCP. *PpACO3* expression appeared unaffected by external ethylene manipulation. Among the two “Early” *ACO*, *PpACO5* followed System 1 regulation, showing significant induction when ethylene signaling was inhibited, whereas *PpACO2* responded positively to Ethrel.

Taken together, although some “Late” and “Early” ethylene biosynthesis genes followed the expected System 2 and System 1 patterns, others displayed atypical regulation. This suggests that ethylene biosynthesis in plum may involve more complex, temporally specific regulatory mechanisms, with additional factors beyond ethylene potentially contributing to the regulation.

### Expression dynamics of positive and negative regulators in ethylene signaling are not strictly associated with their functional roles

**Fig. 4:**
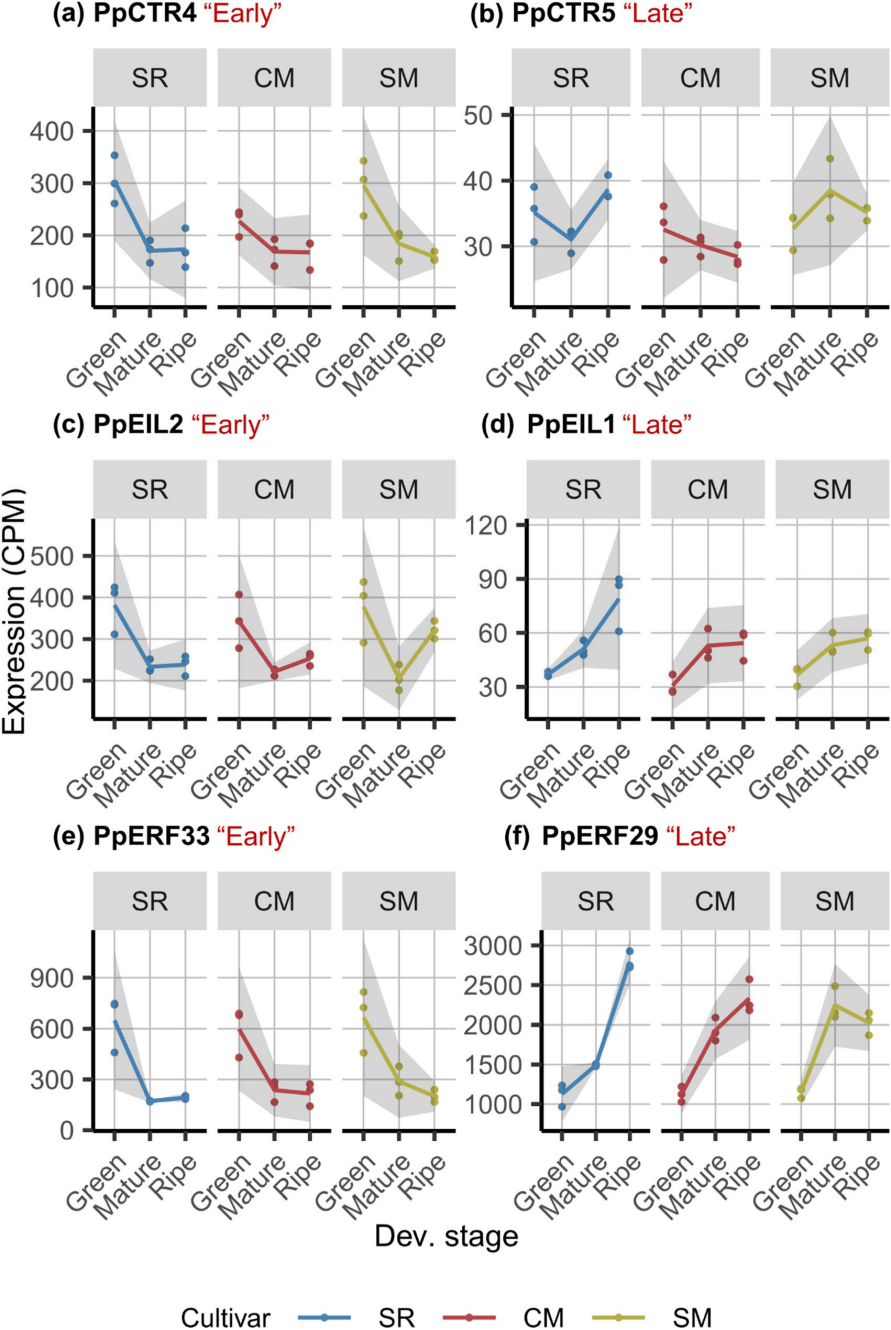
Examples illustrating the diverse expression patterns within positive and negative ethylene signaling regulators, which are not strictly associated with their functional roles. **a**, **c**, and **e** represent “Early” genes, while (**b**), (**d**), and (**f**) represent “Late” genes in the *CTR*, *EIL*, and *ERF* families, respectively. Each facet illustrates expression dynamics in flesh across the “Green”, “Mature”, and “Ripe” stages for ‘Santa Rosa’ (SR), ‘Casselman’ (CM), and ‘Sweet Miriam’ (SM). The grey ribbon around each curve indicates the 95% confidence interval, and red labels (“Late” or “Early”) denote the expression patterns. The comprehensive gene list is provided in Table S2, and the expression plots are in Fig. S2 to S6

Next, we examined the ethylene signaling pathway in SR to evaluate whether the expression dynamics of the negative regulators, *ETR* and *CTR*, consistently differed from those of the positive regulators, *EIN2*, *EIL*, and *ERF*. Except for *ETR*, all gene families contained both “Early” and “Late” genes, irrespective of their classification as positive or negative regulators (Fig. 4). *ETR* expression also exhibited diversity, including “Late” members and those classified in Group 2 (Fig. S2). These findings suggest that the expression dynamics of these genes are not strictly associated with their functional roles in the ethylene signaling pathway but instead exhibit developmental-dependent fluctuations, indicating complex regulatory mechanisms.

### CM and SM share similarities in ethylene-related gene dynamics compared to SR

We examined the expression differences in ethylene-related genes across SR, CM, and SM to test two hypotheses: (1) whether the differences in ethylene-related genes correspond to the ethylene production differences, and (2) whether the expression pattern of ethylene-related genes in suppressed-climacteric CM more closely resembles that of SR or SM.

**Fig. 5:**
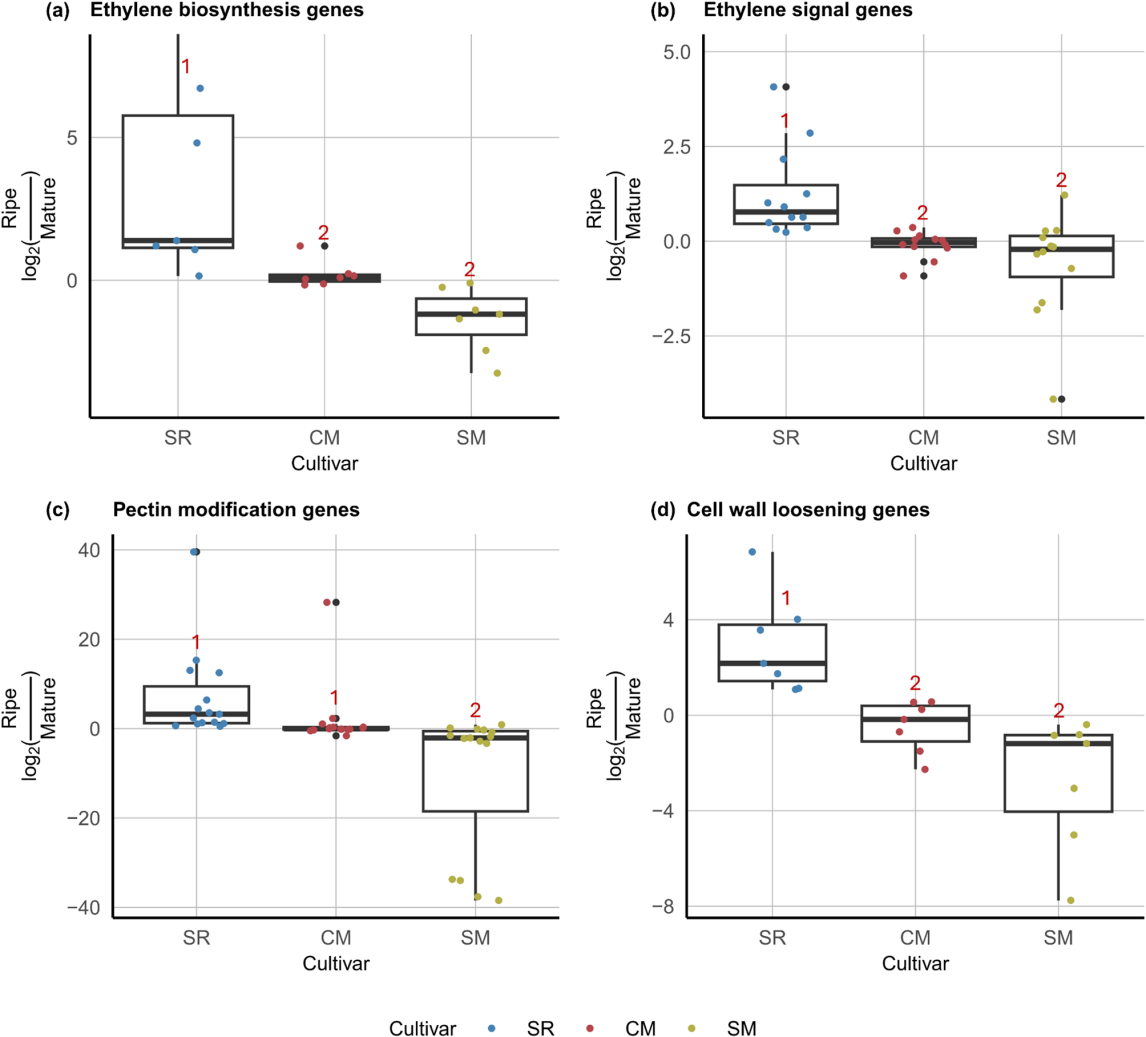
Log ratios of gene expression at the “Ripe” stage relative to the “Mature” stage for “Late” genes reveal significant differences among ‘Santa Rosa’ (SR), ‘Casselman’ (CM), and ‘Sweet Miriam’ (SM). Each point represents a single “Late” gene from (**a**) ethylene biosynthesis, (**b**) ethylene signaling, (**c**) pectin modification, and (**d**) cell wall loosening. Points are colored based on the cultivars. Boxes represent the interquartile range, with horizontal lines indicating the median. Whiskers extend to the minimum and maximum values within 1.5 times the interquartile range. Numbers above the boxes were generated using the Tukey method for multiple pairwise comparisons, with different numbers indicating statistically significant differences (*p* < 0.05)

For the ethylene biosynthesis genes in the *ACS* and *ACO* families, we found that “Early” genes exhibited similar expression patterns across all three cultivars, while “Late” genes showed considerable variation (Fig. 2). Specifically, SR displayed a sharp induction between the “Mature” and “Ripe” stages, whereas CM and SM showed more moderate or declining trends. For example, *PpACO1* expression increased sharply in SR but remained stable in CM and decreased in SM. To quantify these differences, we calculated the log ratio of gene expression at the “Ripe” stage relative to the “Mature” stage for each “Late” gene across three cultivars. As shown in Fig. 5a, all ethylene biosynthesis genes in SR had positive log ratios, which significantly differed from CM and SM. In contrast, those genes in SM exhibited negative log ratios, reflecting a declining trend. In CM, some genes had positive log ratios, though none exceeded 1.5, while others were negative, placing CM between SR and SM. The difference in log ratios between SM and CM was not significant. These findings indicated that “Late”, but not “Early” ethylene biosynthesis genes align with the ethylene production phenotypes [5], with SR showing a pronounced divergence from CM and SM.

Next, we investigated the differences in ethylene signaling genes across SR, CM, and SM. Notably, the majority of “Early” genes (25 out of 36) exhibited similar expression patterns across all cultivars, consistent with the observation in ethylene biosynthesis genes (Fig. S2-S6). However, 11 genes displayed distinct expression patterns. Among these, the low expression of *PpEIL5*, *PpERF1*, and *PpERF49* may contribute to the observed variation. For genes with moderate expression, *PpCTR3* and *PpERF32* exhibited distinct patterns across all three cultivars. *PpERF34*, *PpERF39*, *PpERF41*, and *PpERF44* showed similar expression dynamics in SR and CM, while SM displayed distinct patterns. CM differed from both SR and SM in *PpERF10* expression, whereas *PpERF50* expression was similar in CM and SM but not in SR.

Similar to the “Late” ethylene biosynthesis genes, “Late” ethylene signaling genes in SR also showed a significantly higher log ratio of gene expression at the “Ripe” stage relative to the “Mature” stages compared to CM and SM, indicating a more pronounced positive ethylene-driven response in SR (Fig. 5b). In contrast, the log ratios in CM ranged from 0.4 to −1.0 and were more similar to those observed in SM. Taken together, both “Late” ethylene biosynthesis and signaling genes display more distinct expression patterns in SR among the three cultivars, consistent with the stronger ethylene-driven regulation in this climacteric cultivar.

### Expression dynamics of “late” softening-related genes across cultivars

Based on our previous study, plum softening rates can be stratified into fast, intermediate-to-slow, and slow, corresponding to climacteric, suppressed-climacteric, and non-climacteric ripening behaviors, respectively [5]. To test whether softening-related genes differ across these groups, we analyzed expression patterns in SR, CM, and SM. Using the same workflow described for ethylene-related gene analyses, we first generated comprehensive lists of pectin modification enzymes (PME, PG, PL, and BGAL) and cell wall loosening proteins (EXP, XTH, and EG) (Table S2). All enzyme groups, except for EXP, are composed of multiple gene families that share the same enzymatic activity. These genes were then categorized based on their expression patterns using hierarchical clustering. As shown in Fig. S7, the expression patterns of softening-related genes were more variable compared to ethylene-related genes. Among the six distinct groups identified, Group 2 and Group 3 corresponded to the “Early” and “Late” gene expression patterns, respectively (Fig. S8). We focused on these two groups as they represented the largest clusters, and the highly expressed genes were predominantly found within these groups.

Similar to ethylene-related genes, “Early” pectin modification genes exhibited similar expression patterns across SR, CM, and SM, with a few exceptions (*PpPME10*, *PpBGAL2*, and *PpBGAL12*). In contrast, “Late” pectin modification genes displayed distinct expression patterns among cultivars (Fig. S9-S12). As shown in Fig. 5c, the log ratio of gene expression at the “Ripe” stage relative to the “Mature” stage differed significantly between SR and SM, as well as between CM and SM. SR exhibited high log ratios, indicating sharp increases in pectin modification gene expression during the transition between the “Mature” and “Ripe” stages, while SM showed mostly negative log ratios and indicated an opposite trend. CM displayed an intermediate pattern with log ratios near 0, suggesting stable expression levels. The results for cell wall loosening genes were consistent with those of the aforementioned genes: Most “Early” genes exhibited similar expression patterns across three cultivars (Fig S13-S15). For “Late” genes, the log ratios were highest in SR, followed by CM, and lowest in SM, indicating a lack of induction of these genes in CM and SM (Fig. 5d). Overall, these trends of the “Late” genes align with previous reports on softening differences among plums with varying climacteric behaviors, where climacteric cultivars exhibited the fastest softening, followed by suppressed-climacteric cultivars, and non-climacteric cultivars softened the slowest [5].

### DGE analysis indicated additional factors related to the climacteric ripening differences

**Fig. 6:**
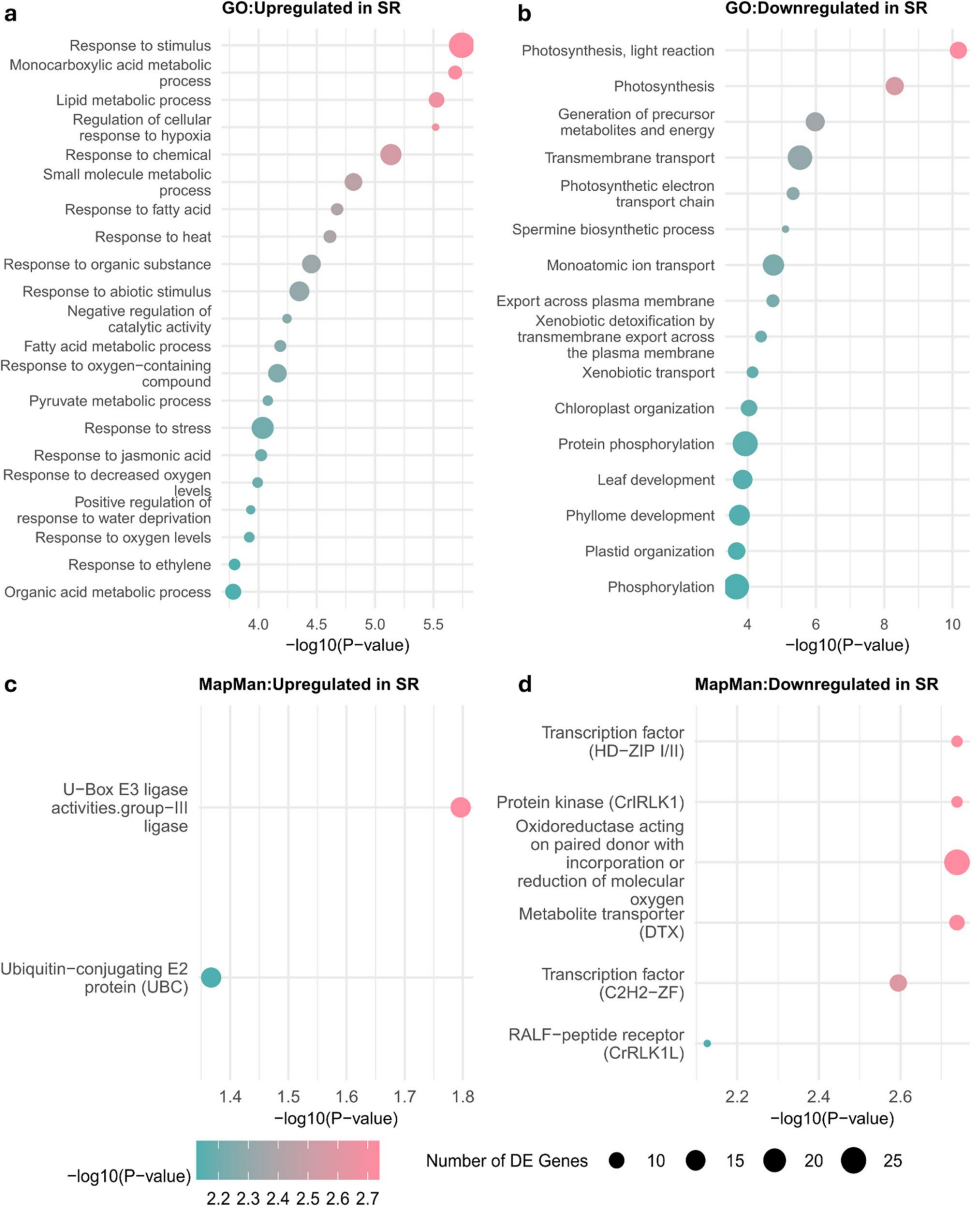
Gene Ontology (GO) (**a**, **b**) and MapMan enrichment analyses (**c**, **d**) of candidate genes exhibiting differential expression (DE) in ‘Santa Rosa’ (SR), ‘Casselman’, and ‘Sweet Miriam’. Differences between the “Mature” and “Ripe” stages, as well as distinct expression patterns across three developmental stages, were examined. Candidate genes were divided into two groups based on whether they were (**a**,** c**) upregulated or (**b**,** d**) downregulated between the “Mature” and “Ripe” stages in SR. Point size represents the number of DE genes. Color indicates *p*-values on a log scale, with pink and green representing smaller and higher *p*-values, respectively

Crosstalk between ethylene and other regulatory factors plays a critical role in ripening behaviors. To explore additional regulatory mechanisms contributing to the substantial differences observed during the later ripening stages across cultivars, we performed differential gene expression (DGE) analysis (see Materials and Methods for details on the criteria used). We identified 2,274 candidate genes, with 1,064 upregulated and 1,210 downregulated during the climacteric stage in SR. Gene ontology (GO) enrichment analysis revealed that a large proportion of enriched GO terms in the downregulated genes were related to photosynthesis and plastid organization (Fig. 6b, Table S4), consistent with the progression of fruit ripening. In contrast, the upregulated genes were enriched in various external and internal response pathways, including response to ethylene (GO:0009723) and jasmonic acid (JA) (GO:0009753) (Fig. 6a, Table S4). Among the 22 genes annotated with responses to ethylene, 12 were involved in ethylene production and signaling. Similarly, seven out of 26 genes annotated with JA response acid were associated with JA biosynthesis and regulation, including *PpJAR1*, which catalyzes the formation of biologically active jasmonyl-isoleucine. *PpJAR1* exhibited distinct patterns across the three cultivars, with higher expression in SR at the “Ripe” stage (Fig. S16). Interestingly, several *ERF*s responding to JA were found, including *PpERF15*, *PpERF18*, and *PpERF21*. Additionally, we identified two *NAC* transcription factors, *PpNAC2* (Prupe.4G279600) and *PpNAC72* (Prupe.4G186800), which belong to a family known to coordinate ripening phenotypes in peach [54].

We also tested for overrepresentation of biological pathways using MapMan. The upregulated genes were overrepresented by two MapMan terms, both related to ubiquitin conjugation activities (Fig. 6c). Six MapMan terms were enriched in the downregulated genes (Fig. 6d), including HD-ZIP I/II and C2H2-ZF transcription factors, which are involved in regulating hormones such as ethylene, JA, and ABA [55, 56]. Collectively, our DGE and enrichment results suggest that other hormones, particularly JA, may also contribute to the differences in climacteric ripening.

### “Late” ethylene- and softening-related genes cluster into two gene modules, each significantly correlated with phenotype differences at the “Ripe” stage

**Fig. 7:**
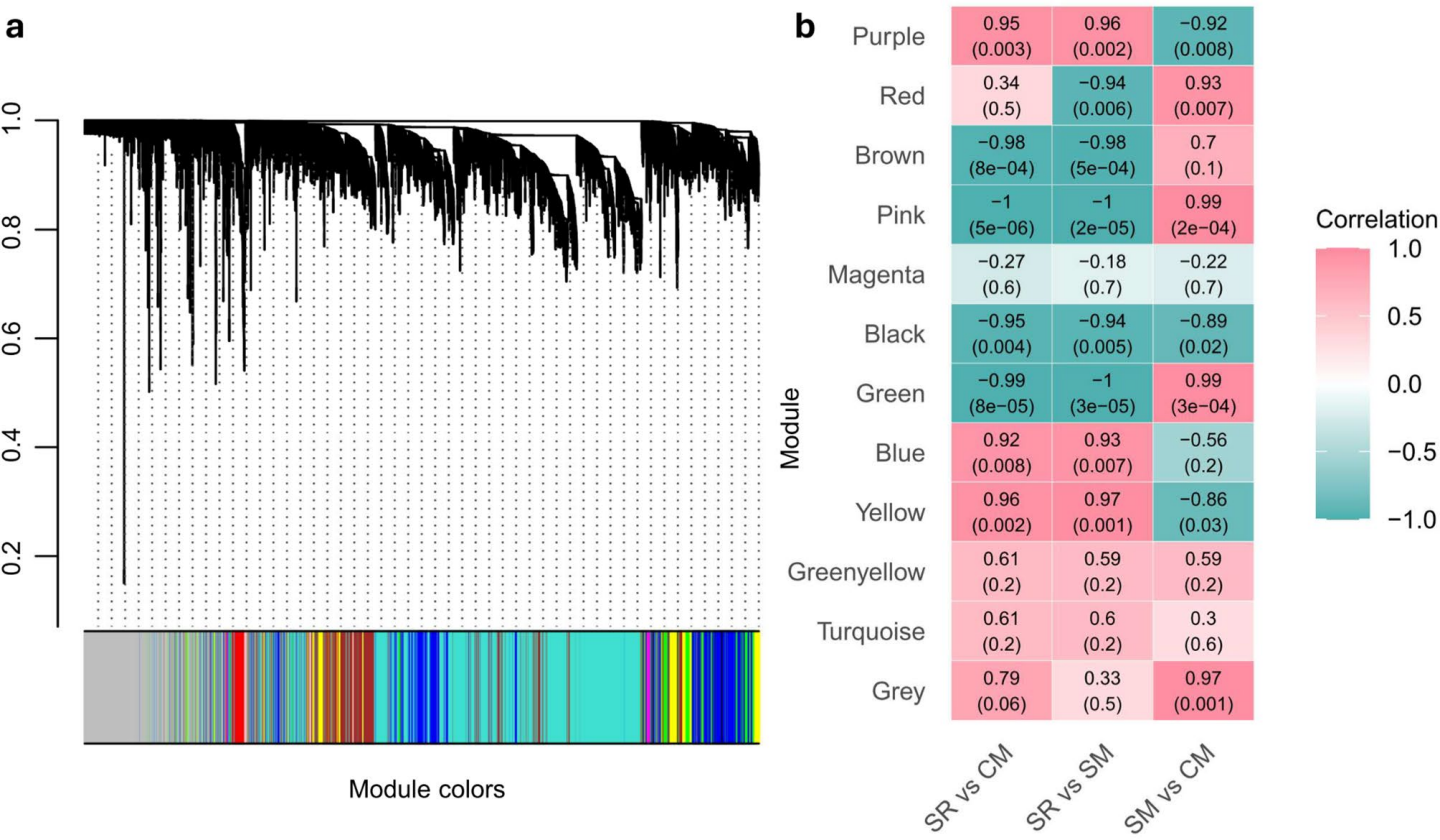
Weighted gene co-expression network analysis (WGCNA) showing the correlation between gene modules and differences among ‘Santa Rosa’ (SR), ‘Casselman’ (CM), and ‘Sweet Miriam’ (SM) at the ‘Ripe’ stage. **a** WGCNA grouped genes into 11 modules of highly correlated expression patterns. Different colors represent distinct modules. The Grey module contains unassigned genes. **b** Heatmap showing correlations between modules and pairwise differences among cultivars. Row names represent modules, and column names represent pairwise comparisons. Numbers in the heatmap indicate Pearson correlation coefficients, with *p*-values in parentheses. Color coding reflects the direction of correlation: pink for positive and green for negative

To further explore factors beyond ethylene influencing ripening differences among the three cultivars, we performed a gene co-expression network analysis using WGCNA, identifying 11 groups of highly correlated genes (“modules”) (Fig. 7a). Notably, a large proportion of “Late” ethylene- and softening-related genes were assigned to the Blue and Brown modules, including 8 of 18 ethylene-related genes and 12 of 22 softening-related genes. *PpJAR1*, identified in the DGE analysis, was assigned to the Brown module. Furthermore, significant correlations were observed between the Blue and Brown modules and the differences in SR vs. CM and SR vs. SM, but not CM vs. SM at the “Ripe” stage (Fig. 7b), aligning with our previous findings (Fig. 5).

Next, we identified hub genes in the Blue and Brown modules by selecting the top 10 genes with the highest connectivity (Table S5). Several hub genes in the Brown module were transcription factors, including *PpNAC2*, which was also identified in our DGE analysis. In the Blue module, hub genes *PpPED1* (Prupe.1G003300), *PpJID1-1* (Prupe.1G035800), and *PpJID1-2* (Prupe.1G036000) were linked to JA regulation, with *PpPED1* predicted to be involved in JA biosynthesis [57], while *PpJID1-1* and *PpJID1-2* were predicted as negative regulators [58, 59].

### Validation of target genes using an independent RNA-seq dataset

Our comprehensive analyses provided logical and efficient criteria to narrow the number of candidate genes across different gene families. We focused on “Late” genes with the highest or the second-highest expression within each enzyme group. We selected *PpACS1* and *PpACO1* for ethylene biosynthesis, *PpPME65*, *PpPL1*, *PpPG54*, and *PpBGAL16* for pectin modification, and *PpXTH21* for cell wall loosening. *EG* and *EXP* families were excluded due to the low expression levels of “Late” genes within these groups. Additionally, we included *PpJAR1*, *PpNAC2*, *PpPED1*, *PpJID1-1*, and *PpJID1-2* from DGE and hub gene analyses.

To evaluate whether these target genes exhibit consistent expression patterns under different conditions, we reanalyzed publicly available RNA-seq data from NCBI SRA database. Specifically, we downloaded and processed raw sequencing data from Farcuh et al. (2017) [29] using our independently developed pipeline. This dataset included SR and SM at the “Ripe” stage and was collected prior to our RNA-seq experiment. Except for *PpACS1* and *PpACO1*, none of the genes listed below were examined in the original publication.

**Fig. 8:**
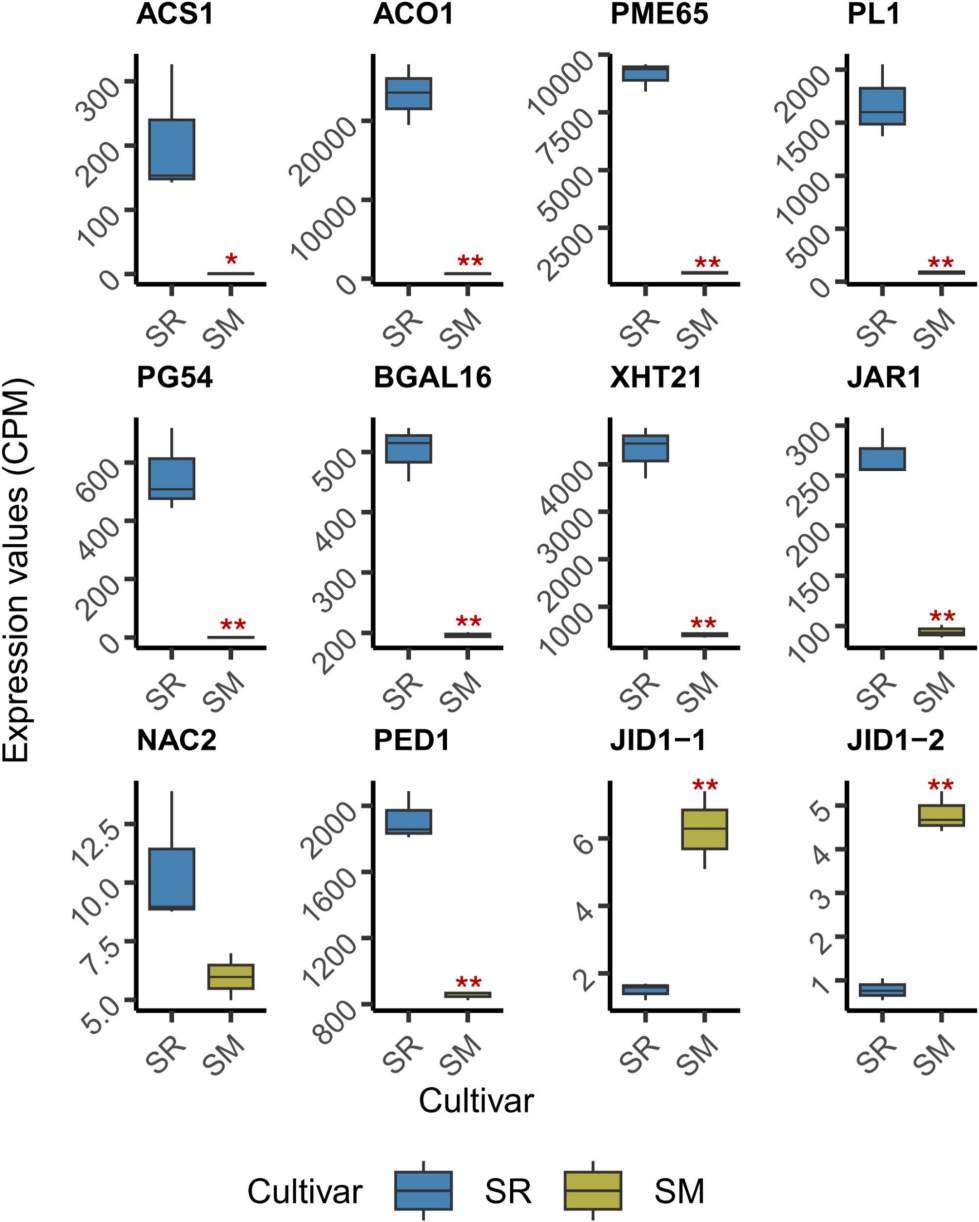
Expression differences of target genes between ‘Santa Rosa’ (SR) and ‘Sweet Miriam’ (SM) in an independent dataset are consistent with our RNA-seq results. Raw sequencing data from Farcuh et al. (2017) were reanalyzed using our independently developed pipeline, targeting a different set of genes than the original study. Boxes, colored by cultivar, represent the interquartile range, with horizontal lines indicating the median. Whiskers extend to the minimum and maximum values within 1.5 times the interquartile range. Asterisks indicate significance levels (*p < 0.05, **p < 0.01) based on *t*-tests comparing SM to SR

As shown in Fig. 8, the expression differences in these genes within Farcuh et al.’s dataset were consistent with our RNA-seq results (Fig. 2 and Fig. S2–S15, summarized in Fig. S16). SR exhibited significantly higher expression than SM for all genes, except for *PpJID1-1* and *PpJID1-2*, which showed the expected reversed trends. These findings support the temporal extrapolation of target gene expression patterns as the two experiments were conducted in different years.

### qPCR validates and extrapolates target gene findings to additional cultivars

Next, we tested whether our findings could be generalized to cultivars beyond SR, CM, and SM by qPCR. To this end, we included ‘Friar’ (FRR) and FT as climacteric plums, and LSR and Ang as suppressed-climacteric plums during the 2024 season, conducted seven years after the RNA-seq sample collection and involved some localization and handling differences (see Materials and Methods for details). Thus, this analysis also allowed us to examine whether our findings could be extrapolated to broader conditions.

**Fig. 9:**
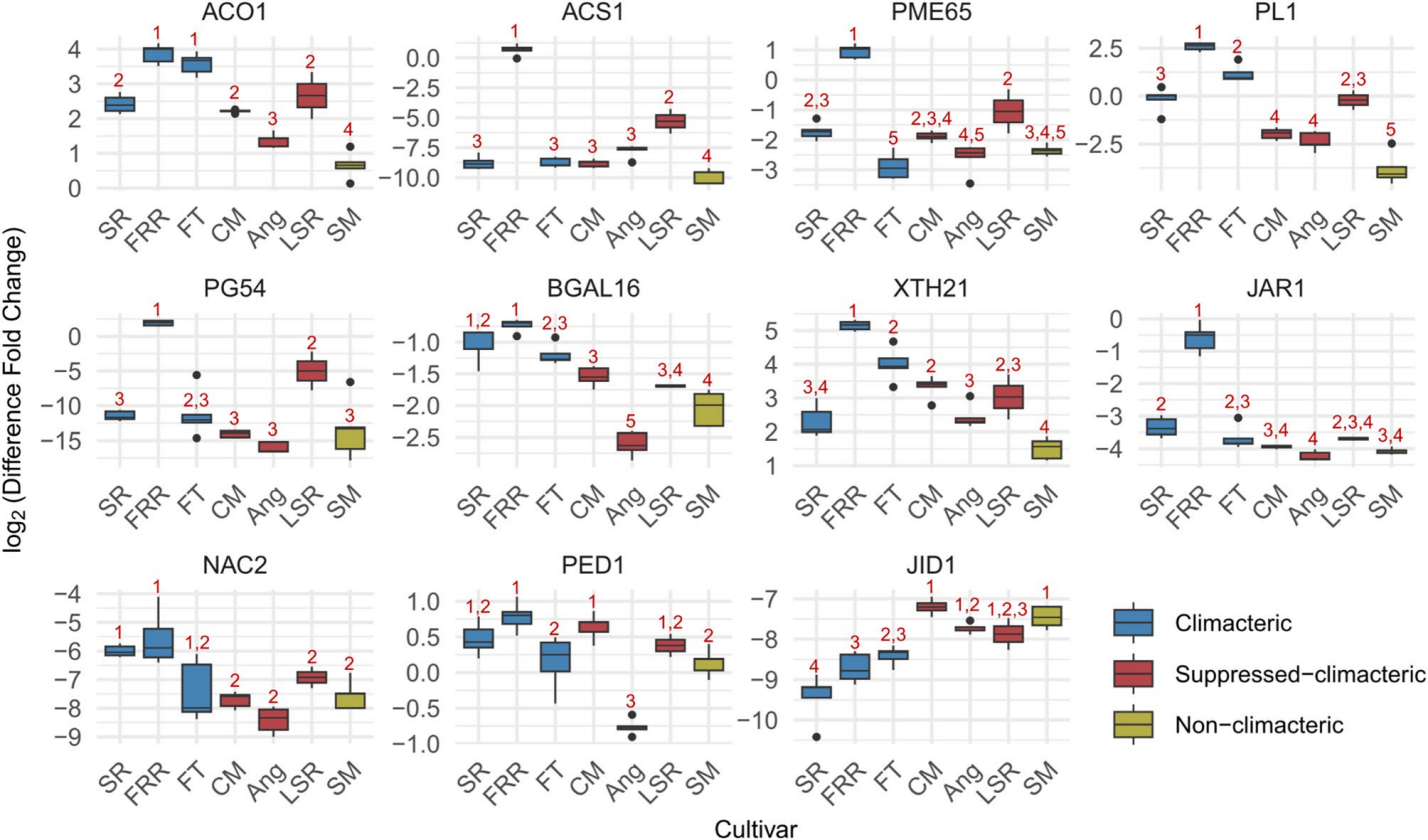
qPCR analysis of target gene expression in additional climacteric and suppressed-climacteric plum cultivars during the 2024 season. Boxes are colored according to the climacteric ripening behaviors corresponding to ‘Santa Rosa’ (SR), ‘Casselman’ (CM), and ‘Sweet Miriam’ (SM). Each box represents the interquartile range, with horizontal lines indicating the median. Whiskers extend to the minimum and maximum values within 1.5 times the interquartile range. Numbers above the boxes were based on the Tukey method for multiple pairwise comparisons, with different numbers indicating statistically significant differences (*p* < 0.05)

Most target genes showed expression trends consistent with the RNA-seq results across climacteric, suppressed-climacteric, and non-climacteric plums (Fig. 9). Notably, target gene expression in SM generally differed from climacteric plums, except for *PpPME65*, *PpPG54*, and *PpPED*1. Interestingly, FRR, the most distantly related cultivar examined [5], exhibited stable expression differences compared to suppressed- and non-climacteric plums for all target genes.

However, the differences across climacteric categories were less distinct than in the RNA-seq results, even among SR, CM, and SM. Variations in target gene expression within the same climacteric category made the differences appear more continuous rather than discrete, blurring the boundaries between categories. Interestingly, the qPCR results also suggested that some target genes may be more robust across varying conditions. For example, *PpACO1* consistently showed larger differences across all three climacteric categories, while *PpACS1*, despite being in the same pathway, exhibited much subtler differences in qPCR. Based on these findings, we propose *PpACO1*, *PpPL1*, *PpBGAL16*, *PpNAC2*, and *PpJID1* as robust candidates for future investigations due to their consistency across conditions and climacteric categories.

## Discussion

This study investigates the underlying transcriptional differences among plums with distinct climacteric ripening behaviors. The availability of closely related cultivars provides a valuable opportunity to identify genes with expression patterns corresponding to the sharp variations in softening across climacteric categories described by Minas et al. [5], determine whether suppressed-climacteric plums are more similar to climacteric or non-climacteric plums, and assess whether findings from a few representatives are generalizable. To this end, we developed a workflow that offers a more comprehensive investigation than previous plum studies and identified several ethylene- and softening-related genes that consistently differ among plums with varying climacteric ripening behaviors. This approach began with comparisons across SR, CM, and SM, and validated findings using publicly available datasets from different years, as well as qPCR data from a broader range of cultivars collected in a subsequent year. To address the challenges of genomic data from species with limited prior experimental evidence, we established a novel database integration procedure and leveraged developmental information to classify genes into “Early” and “Late” groups based on their expression patterns. This workflow refines target gene identification while minimizing the risk of “cherry-picking” specific genes. Such an approach is critical, as softening is a complex physiological process involving crosstalk among numerous genes. Modifying multiple genes provides an opportunity to further improve textural characteristics [10, 11, 60], underscoring the importance of efficiently identifying and targeting multiple softening-related genes for follow-up gene-editing experiments and applications.

### Incorporating developmental stage information enhances insights into gene expression patterns and functional specialization

Clustering genes based on their expression patterns across developmental stages not only streamlined the analysis process but also provided insights into their underlying biological roles. This approach is particularly valuable for large gene families, where different homologs may exhibit spatial or temporal functional specialization. Our strategy also demonstrates that including three developmental stages offers considerably more information compared to previous plum RNA-seq experiments with fewer than two timepoints, where only linear trends could be detected. Using this method, we found “Late” genes as good candidates related to the ethylene and softening differences across climacteric categories in plums, consistent with the physiological distinctions between categories.

Additionally, this method allowed our ethylene-related analyses to uncover previously unrecognized complexities in plum. We found that not all “Early” and “Late” *ACS* and *ACO* genes aligned with the typical definition of System 1 and System 2 ethylene biosynthesis, which is majorly established in tomato. For example, *PpACO3* and *PpACO7* showed higher expression at the “Ripe” stage in SR but did not respond to 1-MCP or Ethrel as expected for System 2 genes. This suggests that some ethylene biosynthesis genes are not directly regulated by ethylene but modulated by other factors, allowing for nuanced regulatory adjustments. Unlike tomato, which follows a single sigmoid developmental curve [61], plum undergoes a double sigmoid developmental pattern [62]. Thus, the differences in ethylene regulation could reflect physiological distinctions between species. Another possibility lies in differences in the central feedback loop regulating ripening, with *MADS* genes being central in tomato and *NAC* genes playing a pivotal role in peach [54]. Thus, applying definitions derived from one species to another requires careful adaptation to account for species-specific traits.

Clustering analysis also sheds light on the functional differentiation among softening genes. For instance, over one-third of the genes in the *PME*, *XTH*, and *EG* families detected in our samples were classified as “Early” genes, with several exhibiting high to moderate expression levels, compared to less than a quarter of pectinase genes. The higher proportion of “Early” *PME* genes aligns with their role in de-esterifying pectin, a necessary step for subsequent pectin hydrolysis by PG and PL [63]. On the other hand, the temporal differences observed in *XTH* and *EG* expression compared to pectinase genes suggest an ordered sequence of cell wall modification processes and potential crosstalk among enzyme groups.

### Variation in transcriptional regulation suggests that the boundaries among three climacteric categories are not discrete and are influenced by environmental factors

Although extensive phenotyping data document differences among climacteric, suppressed-climacteric, and non-climacteric plums [5], it remains unclear whether suppressed-climacteric plums exhibit transcriptional regulation more similar to climacteric or non-climacteric plums. Previous studies on SR-related cultivars focused solely on SR and SM [29–32]. El-Sharkawy et al., on the other hand, compared a climacteric cultivar with two suppressed-climacteric cultivars from a different plum material system, identifying differences in ethylene and auxin pathways [64–70]. However, the genetic relationships among cultivars in these studies remain unclear, and no high-throughput experiments were conducted. To the best of our knowledge, this study is the first to systematically investigate expression differences across all three climacteric categories.

The log ratios of gene expression at the “Ripe” stage relative to the “Mature” stage provide a quantitative measure of the differences in “Late” genes among SR, CM, and SM. We observed CM exhibited greater similarity to SM (Fig. 5), possibly due to the low ethylene production characteristic of both suppressed- and non-climacteric plums. However, qPCR results indicated that the boundaries among climacteric categories are not clear-cut. While overall expression trends aligned with expectations, variation across cultivars blurred these distinctions. This is consistent with previously described continuous phenotypic variation: Minas et al. [5] demonstrated that softening rates varied among suppressed-climacteric plums, with LSR softening the fastest, followed by CM, and Ang the slowest. Our findings are also in line with discoveries in melons, where climacteric responses result from the interaction of multiple loci and exhibit continuous distribution [25]. These results emphasize the need for caution when using single cultivars to represent entire climacteric categories and highlight the importance of generalizing findings across a broader spectrum of cultivars.

Additionally, the less pronounced differences among SR, CM, and SM in the qPCR results suggest genotype–environment interactions, as the samples were collected from different locations and seasons with some handling variations. Particularly, temperature is a potential confounding factor, as climacteric plums typically ripen earlier in the hot summer, followed by suppressed-climacteric plums, with non-climacteric plums ripening the latest around early fall. As shown in Table S1, the weekly average temperatures during the 2017 sample collection were 33 °C, 28 °C, and 19 °C for SR, CM, and SM, respectively. Our qPCR data provided valuable insights by mitigating temperature effects, as the plums were harvested under more similar temperature conditions. Consequently, genes exhibiting consistent behaviors across both RNA-seq and qPCR experiments, *PpACO1*, *PpPL1*, *PpBGAL16*, *PpNAC2*, and *PpJID1*, represent robust candidates for future studies. These findings also highlight the importance of collecting data across multiple timepoints, locations, and cultivars to disentangle confounding factors in climacteric ripening studies.

### Complex hormonal and transcription factor crosstalk underlying Plum climacteric ripening

Our DGE results revealed an enrichment of genes annotated with responses to JA (GO:0009753), and our co-expression network analyses identified several hub genes associated with JA regulation. These findings suggest that, in addition to ethylene, JA may contribute to the differences observed among SR, CM, and SM. Emerging evidence shows interactions between ethylene and JA, with JA generally regarded as an activator of ethylene biosynthesis genes in various fruits, including tomato [71, 72], apple [73, 74], and mango [75]. However, JA’s role in plum remains inconclusive. Khan and Singh [95] reported that postharvest MeJA application induced ethylene production, increased *ACS* and *ACO* activity, and promoted softening. Conversely, other studies demonstrated that while pre-harvest MeJA application generally enhanced ethylene production, it increased fruit firmness [76–79]. These variations in MeJA effects may depend on cultivar differences, developmental stage at treatment, MeJA concentration, and application timing (pre- vs. post-harvest) [79–81]. The internal JA dynamics in plum remain uncharacterized, which is needed for further understanding JA’s role in climacteric ripening.

In addition to JA-related genes, several transcription factors were identified in our study, including *NAC*s. Among them, *NAC2* was detected in both DGE and co-expression network analyses. The *NAC*s is shown to play a critical role in System 2 ethylene biosynthesis in species without recent whole-genome duplication, including peach [54]. Given the high collinearity between the plum and peach genomes [82], it is likely that a similar mechanism operates in plum. Current research in peach demonstrates the versatile roles of *NAC*s in fruit ripening, including the regulation of volatiles, cell elongation, cell wall degradation, sugar accumulation, anthocyanin production, and ethylene biosynthesis [83–87]. *NAC*s also respond to phytohormones and a recent study revealed that MeJA enhances ethylene synthesis in kiwifruit through *NAC*s [88]. The importance of *NAC*s suggests a potential evolutionary or breeding trajectory for stone fruits, where mutations in *NAC*s or their binding sites on ethylene-related genes could enable transitions across climacteric categories. Taken together, our identification of JA-related genes and *NAC*s underscores the complexity of hormonal interactions in plum ripening.

### Streamlined data integration strategy for comprehensive analysis of enzyme groups

High-throughput sequencing enables the simultaneous study of numerous genes, such as all members within an enzyme group. It is particularly valuable for organisms with limited experimental evidence, relying on homology-based functional predictions. However, even in tomato, a well-studied fruit model, some *ACS* homologs were only recently characterized [8]. Similarly, we identified several ethylene-related genes not previously reported in stone fruit studies (Table 1). To fully exploit the advantage of high-throughput sequencing, strategies for comprehensive gene family identification are required.

Recent studies on peach softening genes started to address the need for comprehensive gene identification. A commonly used approach involves Hidden Markov Model (HMM) searches using HMMER [89] to identify genes with target domains responsible for specific enzyme activities. Phylogenetic trees are then constructed to elucidate the evolutionary relationships [13, 15, 17, 18]. However, such *de novo* prediction is time-consuming and requires specialized knowledge of several bioinformatics software.

Our strategy addresses these challenges while offering additional advantages. Functional prediction is streamlined by using InterPro annotations available in Phytozome and Dicots PLAZA. Since InterPro integrates data from multiple sources that use HMM-generated predictions, it eliminates the need for independent protein function predictions [90]. EC number and Pathway ID annotations in Phytozome provide an efficient approach for extracting complete enzyme groups through BioMart [91]. Identified genes can be grouped into families based on homologous relationships from the Integrative Orthology Viewer in Dicots PLAZA, with pre-calculated phylogenetic trees from diverse plant lineages available for detailed analysis, enabling more robust evaluation of evolutionary relationships than user-generated trees based on limited species data. Furthermore, since both databases are actively maintained, the information they provide is reusable and reproducible.

Some plum reference genomes are available in the Genome Database for Rosaceae (GDR) [51]. However, the peach genome offers more complete annotation: GDR lacks EC numbers and MapMan4 information, and GO term annotation coverage is lower, with only 59% of genes assigned GO terms, compared to 75% in the peach annotation in PLAZA. Additionally, the available plum genomes are from Chinese cultivars, which are distantly related to the Santa Rosa-derived cultivars in our study. Given the generally high mapping rates (> 85%, slightly higher with plum genomes), we chose the peach genome for its better annotation.

## Conclusion

Using a comprehensive approach, we found that “Late” genes are associated with softening differences across climacteric, suppressed-climacteric, and non-climacteric plums. Notably, *PpACO1*, *PpPL1*, *PpBGAL16*, *PpNAC2*, and *PpJID1* emerged as robust candidates, consistently differing among climacteric types across multiple experiments. At the transcriptional level, ‘Casselman’ (CM) showed greater similarity to ‘Sweet Miriam’ (SM) than to ‘Santa Rosa’ (SR) in ethylene- and softening-related gene expression. However, when incorporating additional cultivars within each climacteric category, expression variations suggest blurred boundaries among them, emphasizing the need for caution when generalizing findings from a limited set of representative cultivars. Together, this study identifies key candidate genes for future research on climacteric ripening in plum and presents an effective bioinformatic workflow for the comprehensive analysis of relevant gene families.

## Supplementary Information

Supplementary Material 1.

Supplementary Material 2.

Supplementary Material 3.

Supplementary Material 4.

## Data Availability

RNAseq data are available via the NCBI SRA database (BioProject: PRJNA1249002).
