# Supplementary material for: Comprehensive transcriptional analysis of ethylene and softening regulation in plums with distinct climacteric ripening behaviors
Source: BMC Plant Biol. 2025 Jul 12;25:908. doi: 10.1186/s12870-025-06932-w (PMC12255070; doi:10.1186/s12870-025-06932-w)
Supplement: Supplementary file 4 — Supplementary Material 4. [file 12870_2025_6932_MOESM4_ESM.docx]

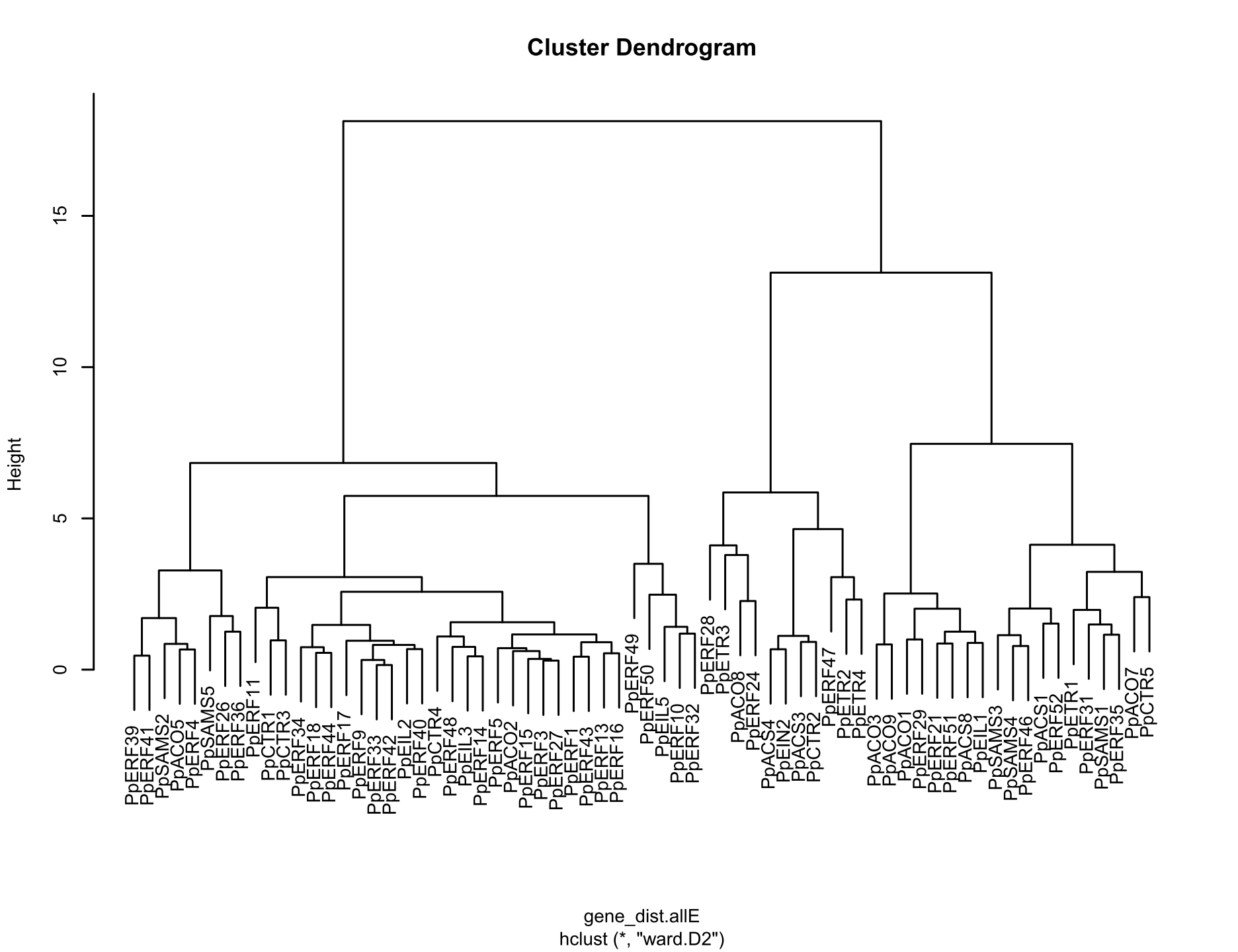


**Fig. S1. Hierarchical clustering of ethylene-related genes based on their expression patterns across three developmental stages in ‘Santa Rosa’.** Identical to Fig. 1, except that this figure displays full gene names.


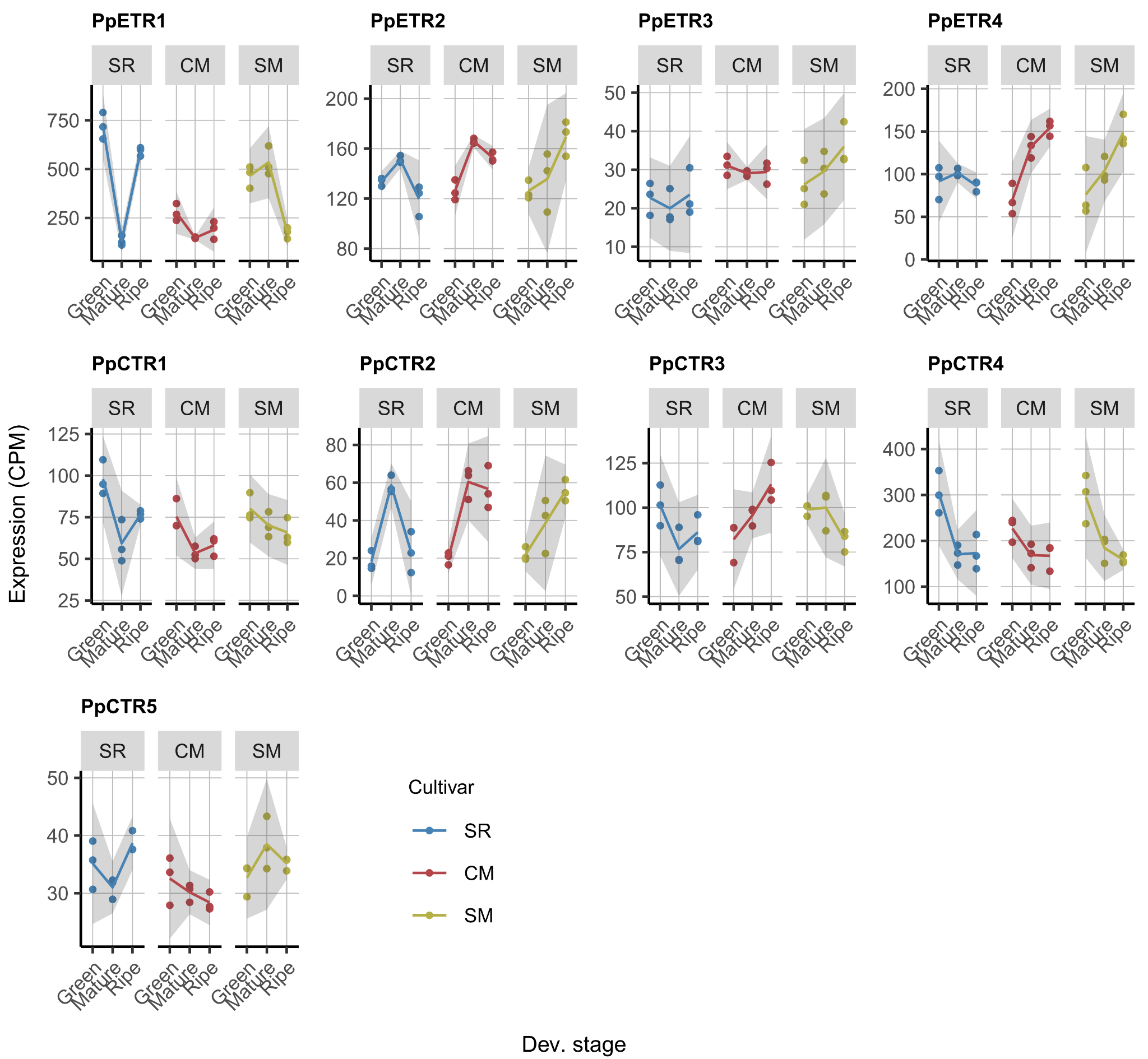


**Fig. S2 Expression patterns of ethylene receptor genes, *ETR* and *CTR* (negative ethylene signaling regulators).** Each facet represents the expression dynamics in flesh across the “Green”, “Mature”, and “Ripe” stages for ‘Santa Rosa’ (SR), ‘Casselman’ (CM), and ‘Sweet Miriam’ (SM). The grey ribbon around each curve indicates the 95% confidence interval.


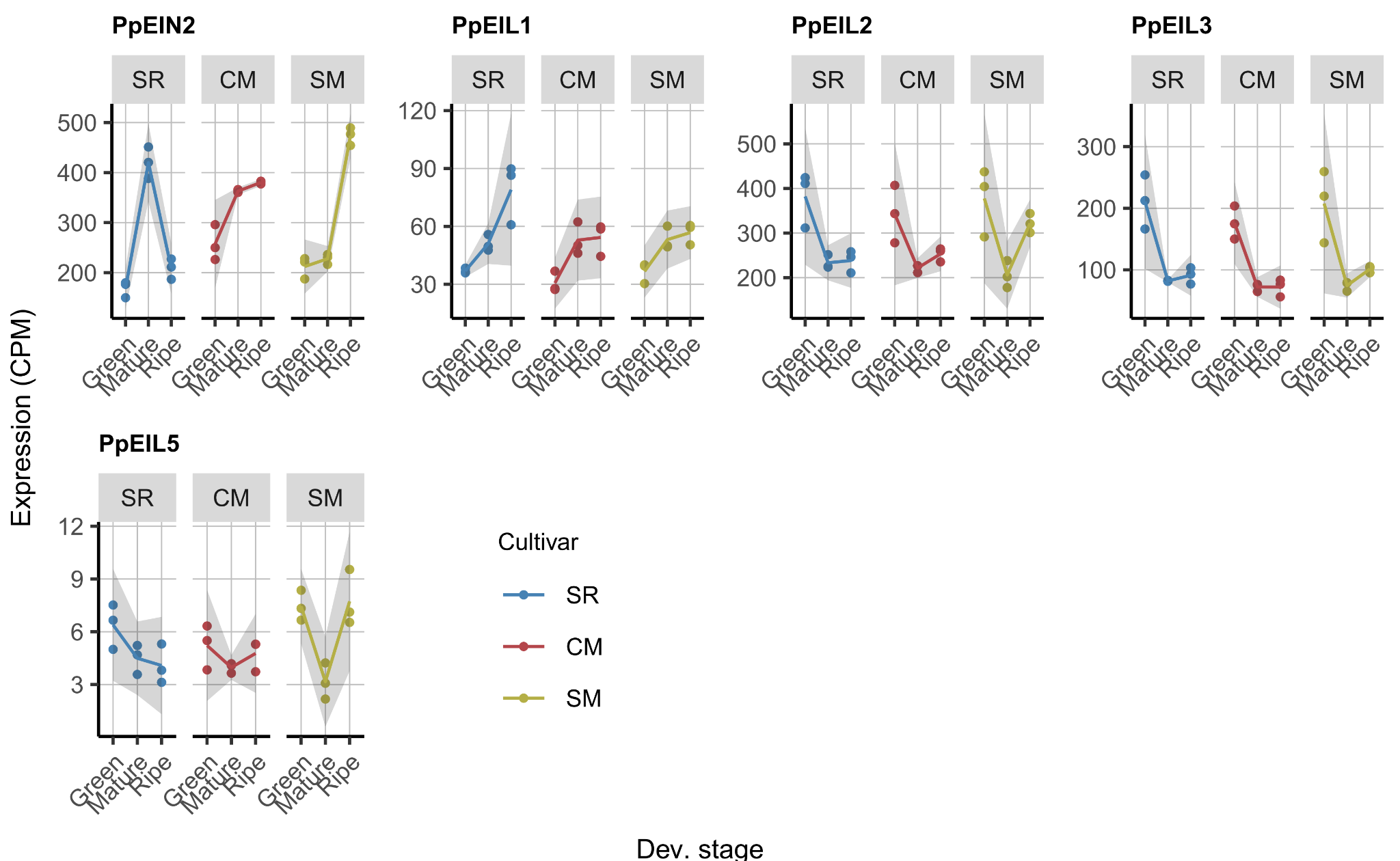


**Fig. S3 Expression patterns of positive ethylene signaling regulators, *EIN2* and *EIL*.** Each facet represents the expression dynamics in flesh across the “Green”, “Mature”, and “Ripe” stages for ‘Santa Rosa’ (SR), ‘Casselman’ (CM), and ‘Sweet Miriam’ (SM). The grey ribbon around each curve indicates the 95% confidence interval.


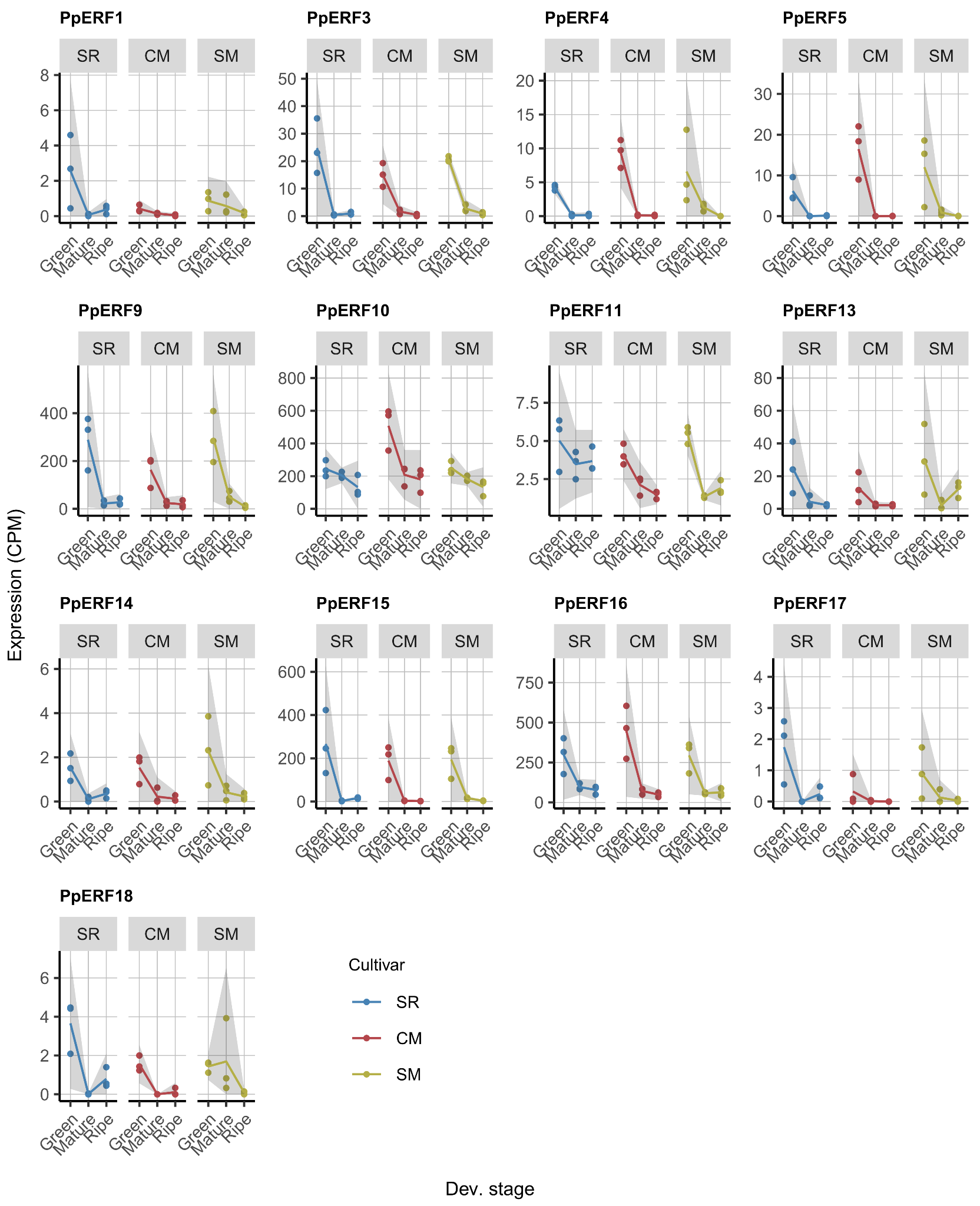


**Fig. S4 Expression patterns of positive ethylene signaling regulators, *ERF* Family 1.** Each facet represents the expression dynamics in flesh across the “Green”, “Mature”, and “Ripe” stages for ‘Santa Rosa’ (SR), ‘Casselman’ (CM), and ‘Sweet Miriam’ (SM). The grey ribbon around each curve indicates the 95% confidence interval.


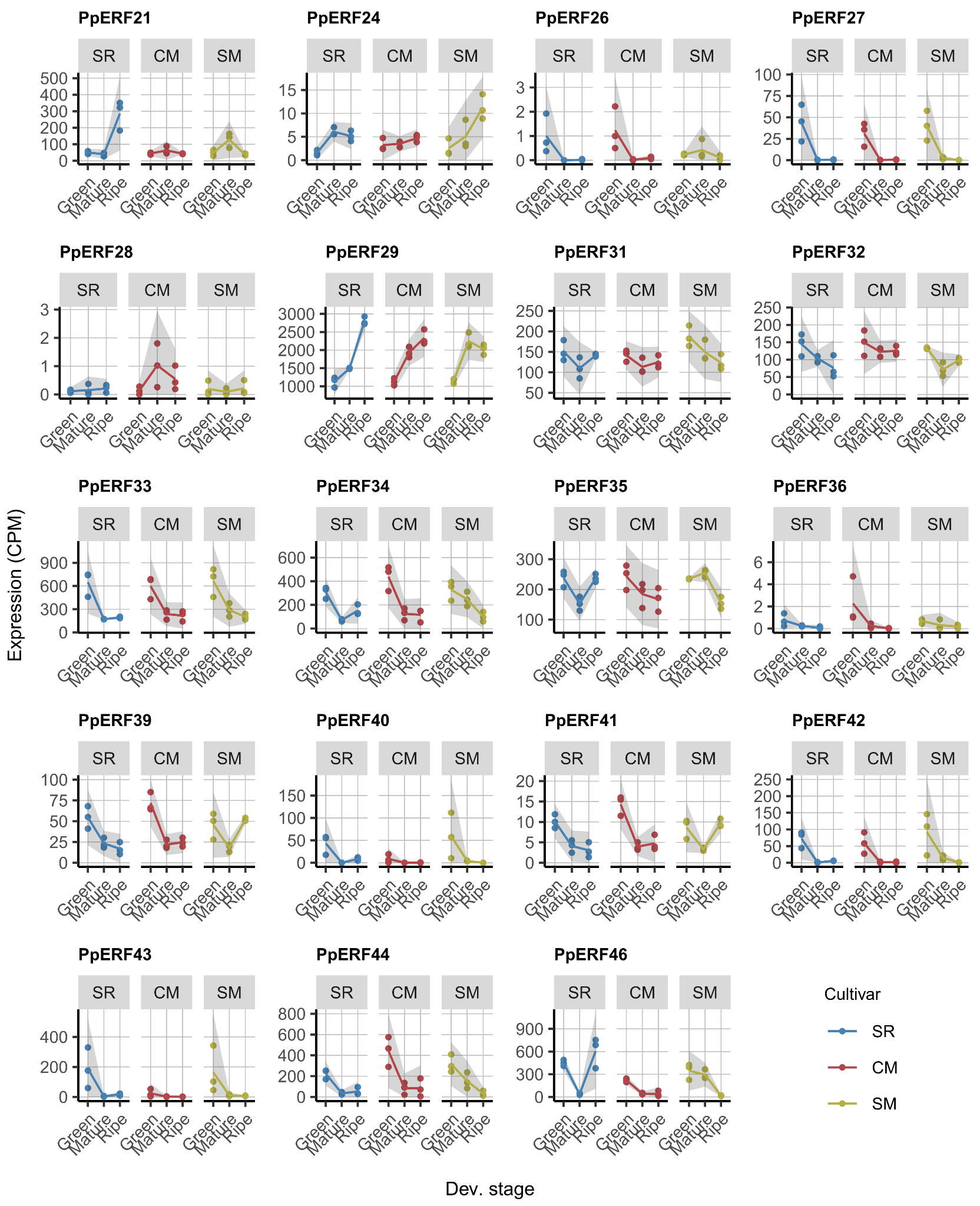


**Fig. S5 Expression patterns of positive ethylene signaling regulators, *ERF* Family 2.** Each facet represents the expression dynamics in flesh across the “Green”, “Mature”, and “Ripe” stages for ‘Santa Rosa’ (SR), ‘Casselman’ (CM), and ‘Sweet Miriam’ (SM). The grey ribbon around each curve indicates the 95% confidence interval.


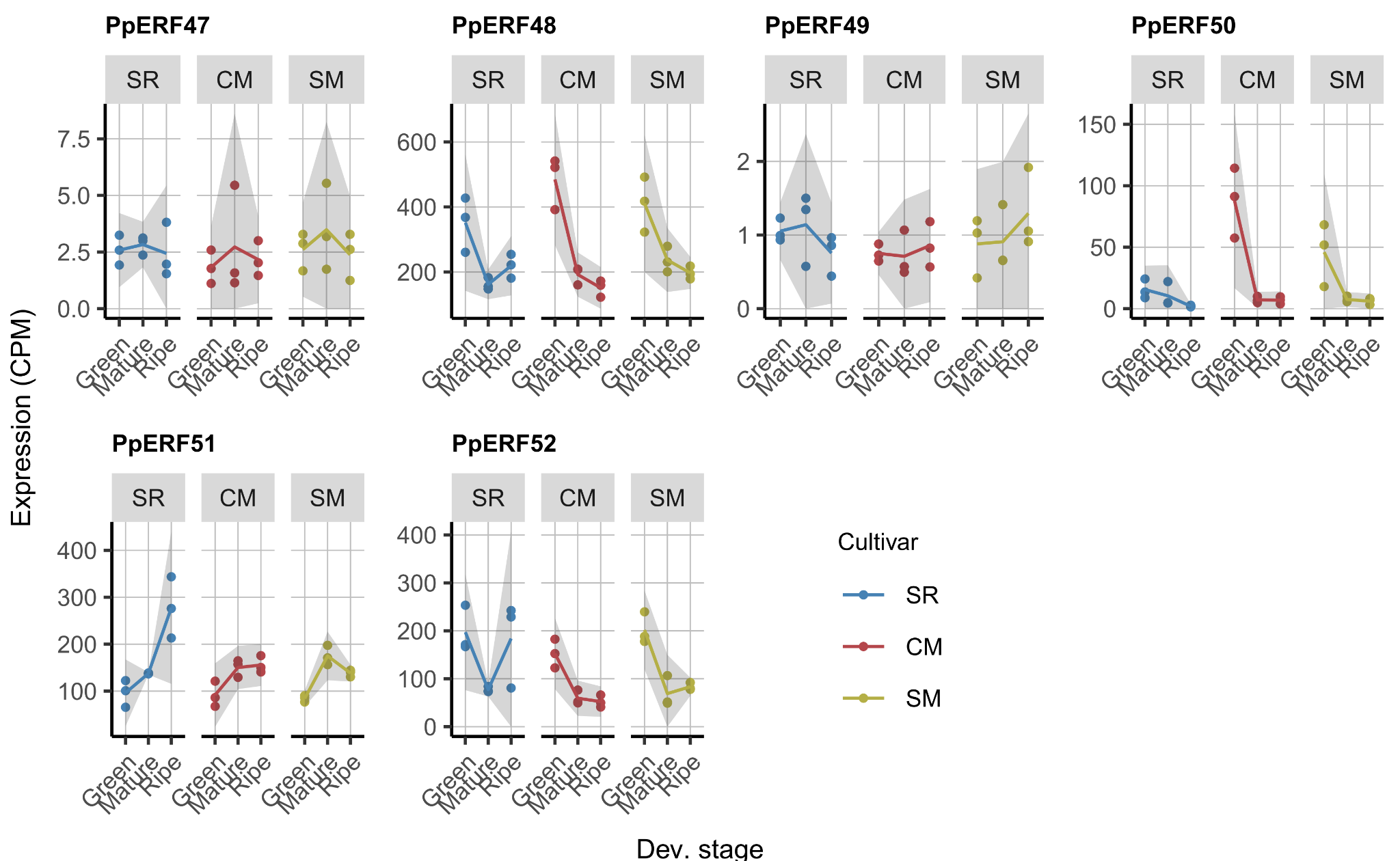


**Fig. S6 Expression patterns of positive ethylene signaling regulators, *ERF* Family 3.** Each facet represents the expression dynamics in flesh across the “Green”, “Mature”, and “Ripe” stages for ‘Santa Rosa’ (SR), ‘Casselman’ (CM), and ‘Sweet Miriam’ (SM). The grey ribbon around each curve indicates the 95% confidence interval.


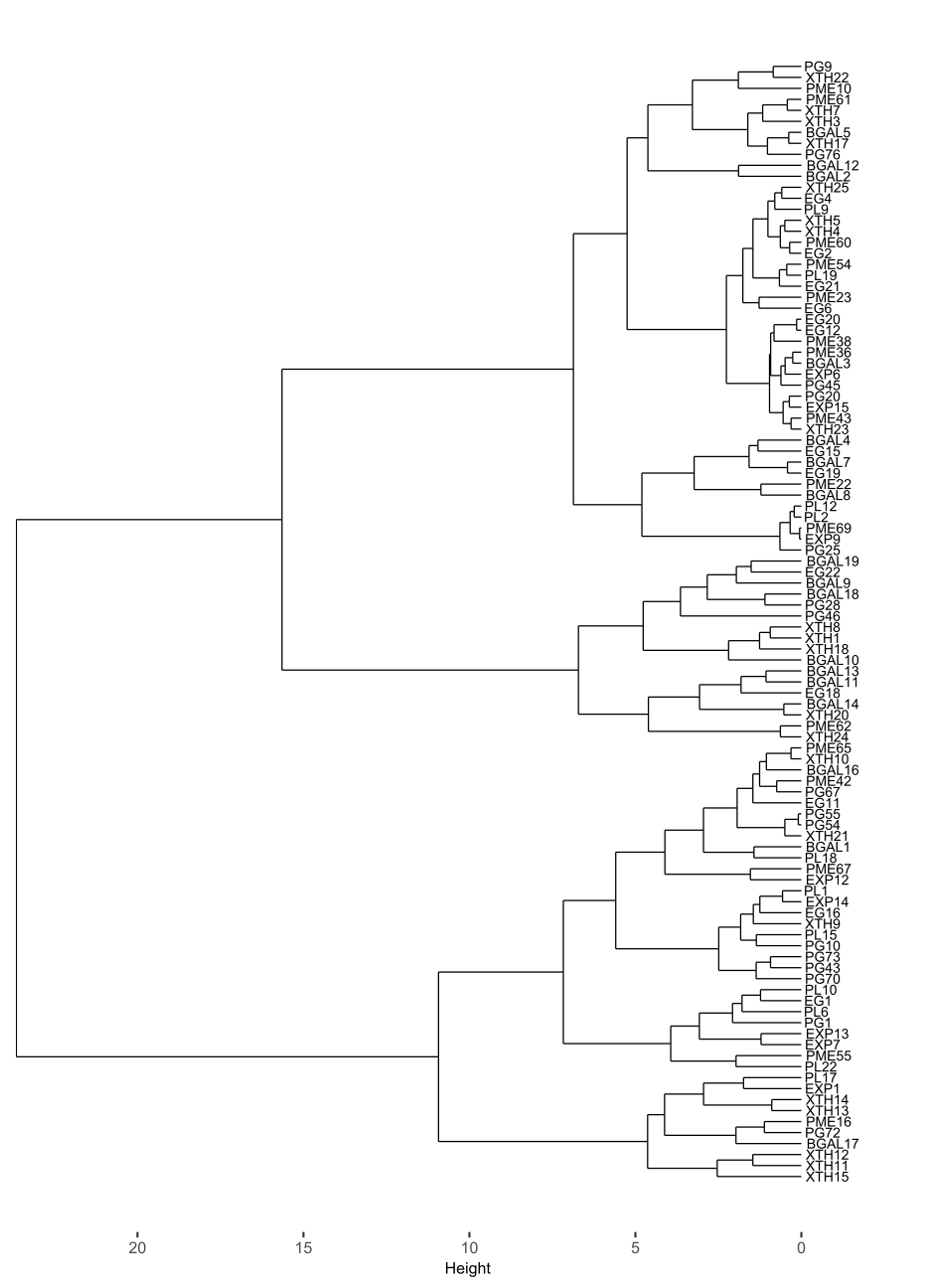


**Fig. S7 Hierarchical clustering of softening-related genes based on their expression patterns across three developmental stages in ‘Santa Rosa’.**


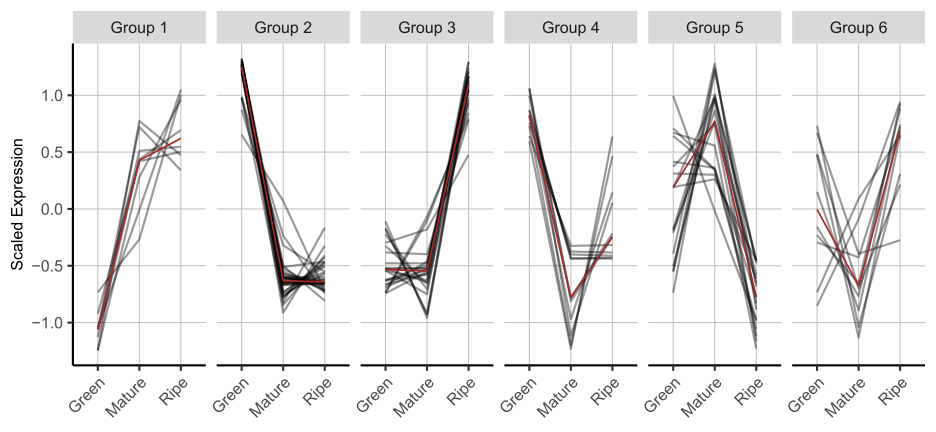


**Fig. S8 Standardized expression dynamics of the softening-related genes within the six groups identified from hierarchical clustering (Fig. S7).** The brown curve represents the median expression trend.


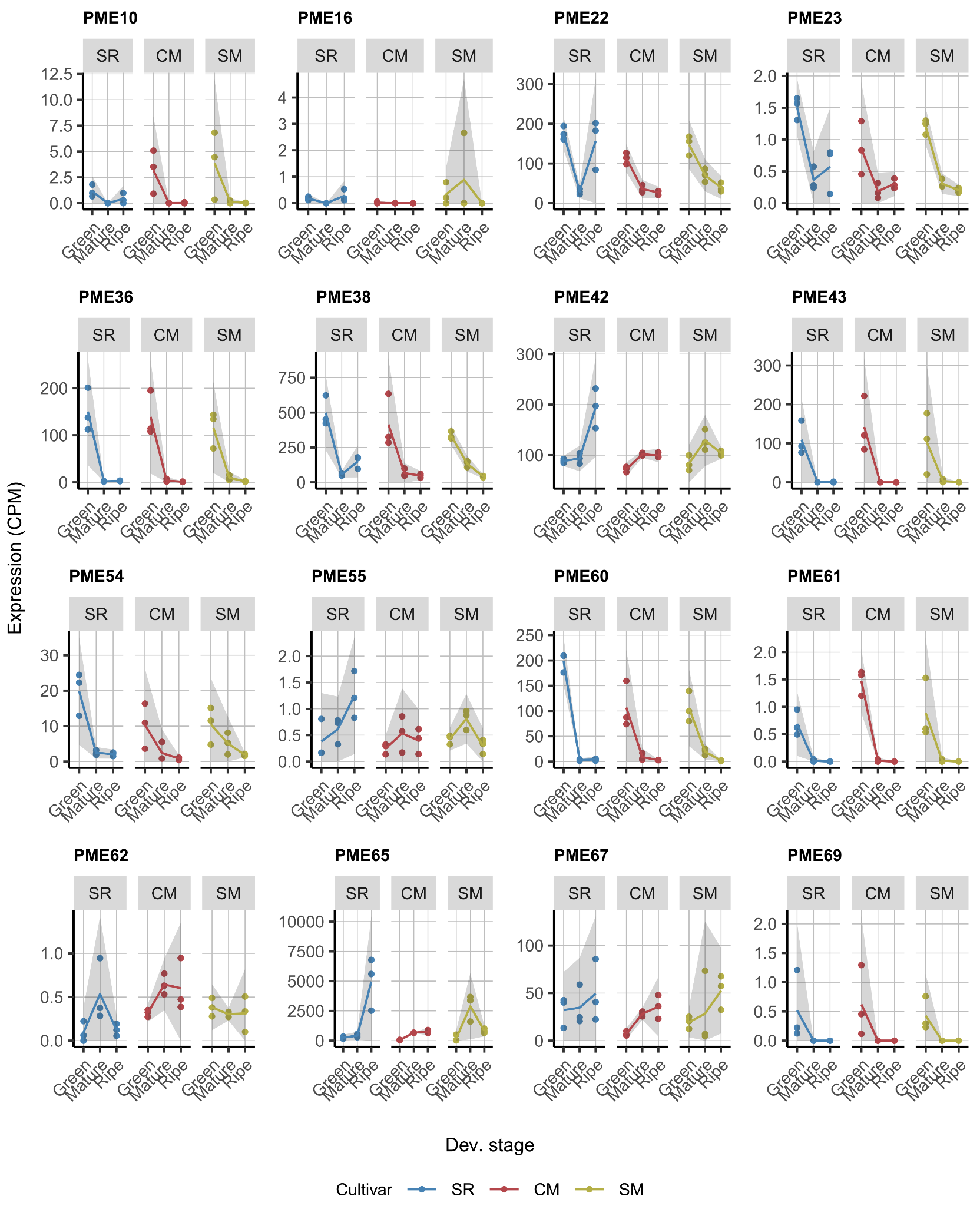


**Fig. S9 Expression patterns of pectin modification genes, *PME*.** Each facet represents the expression dynamics in flesh across the “Green”, “Mature”, and “Ripe” stages for ‘Santa Rosa’ (SR), ‘Casselman’ (CM), and ‘Sweet Miriam’ (SM). The grey ribbon around each curve indicates the 95% confidence interval.


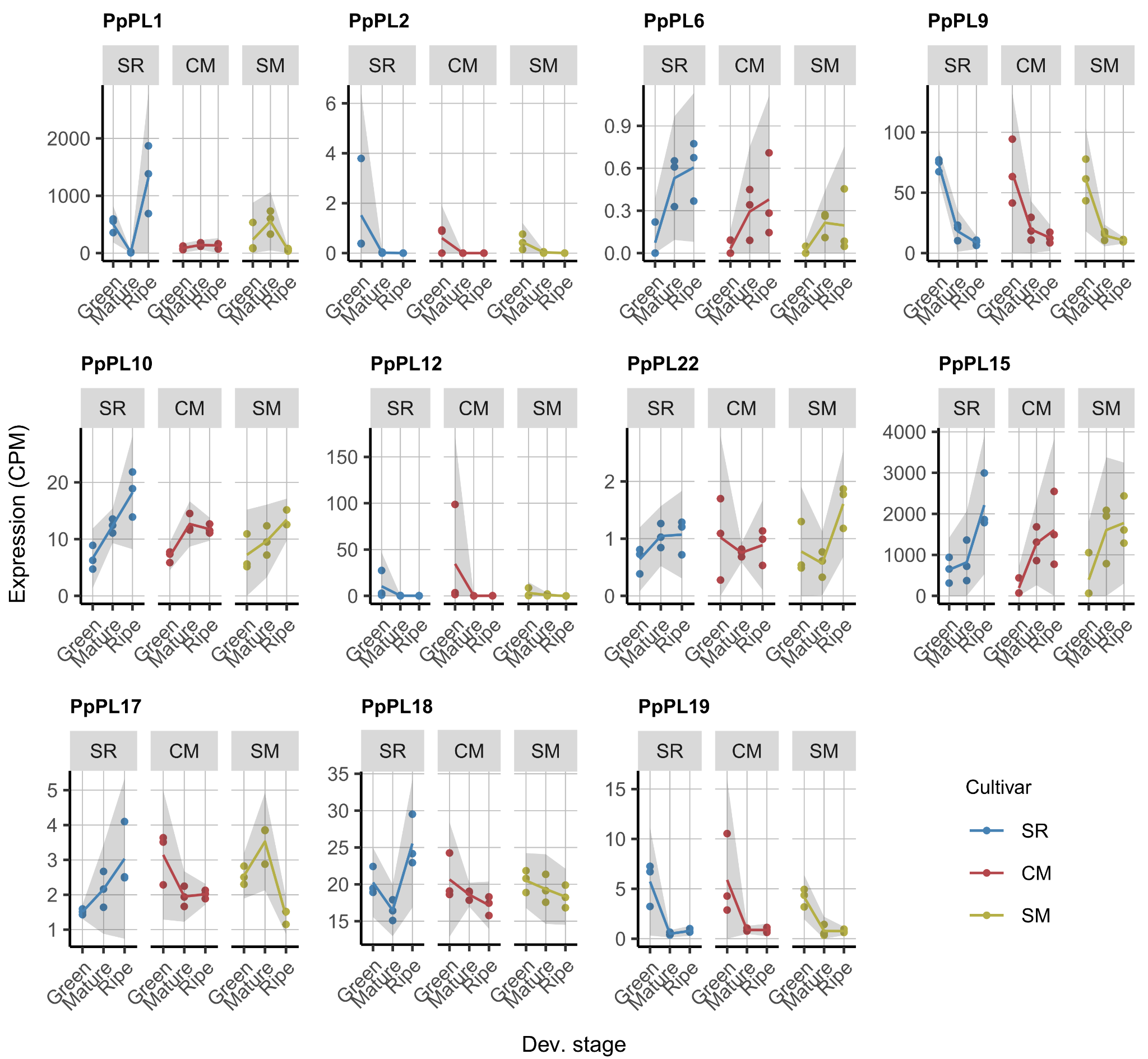


**Fig. S10 Expression patterns of pectin modification genes, *PL*.** Each facet represents the expression dynamics in flesh across the “Green”, “Mature”, and “Ripe” stages for ‘Santa Rosa’ (SR), ‘Casselman’ (CM), and ‘Sweet Miriam’ (SM). The grey ribbon around each curve indicates the 95% confidence interval.


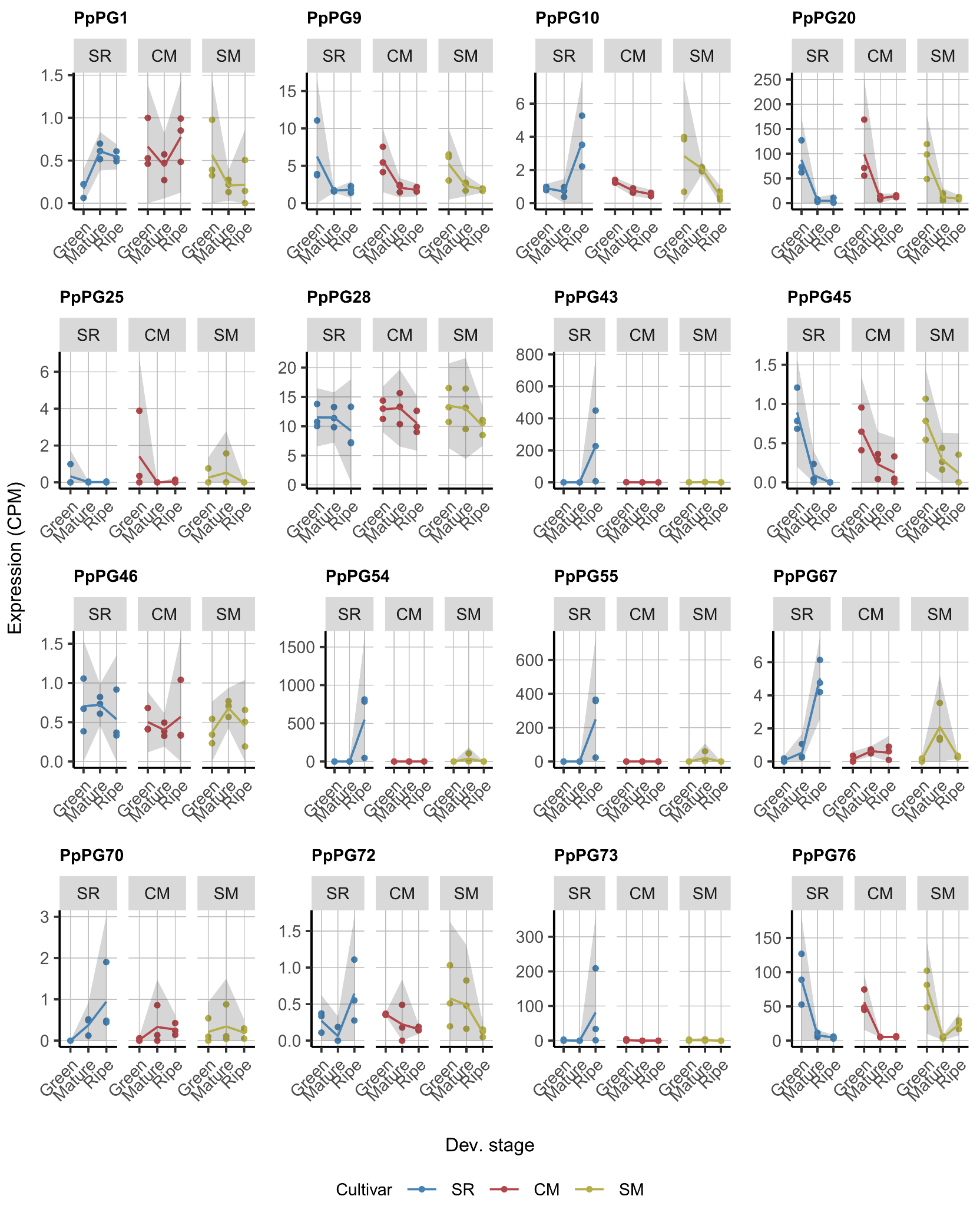


**Fig. S11 Expression patterns of pectin modification genes, *PG*.** Each facet represents the expression dynamics in flesh across the “Green”, “Mature”, and “Ripe” stages for ‘Santa Rosa’ (SR), ‘Casselman’ (CM), and ‘Sweet Miriam’ (SM). The grey ribbon around each curve indicates the 95% confidence interval.

**
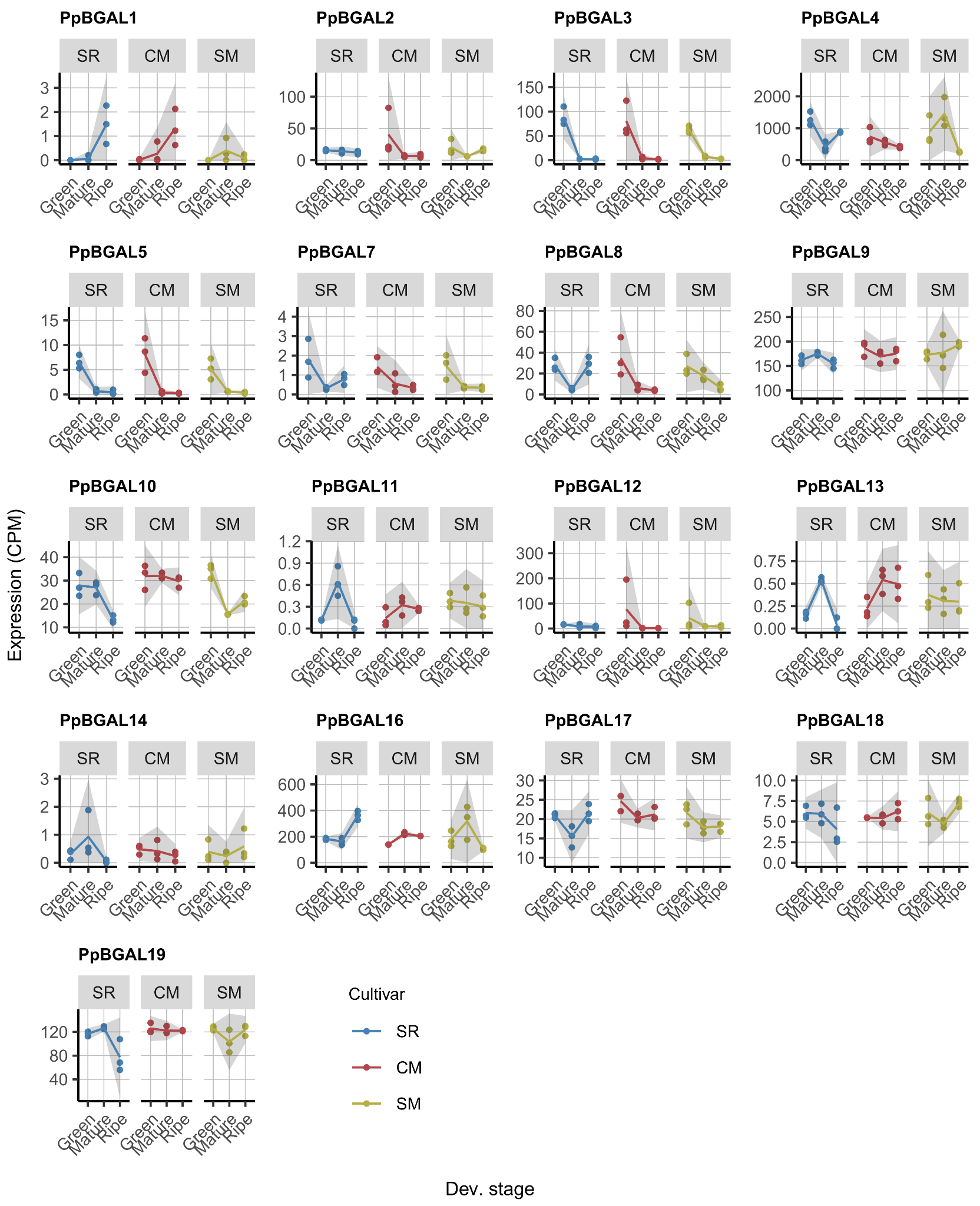
**

**Fig. S12 Expression patterns of pectin modification genes, *BGAL*.** Each facet represents the expression dynamics in flesh across the “Green”, “Mature”, and “Ripe” stages for ‘Santa Rosa’ (SR), ‘Casselman’ (CM), and ‘Sweet Miriam’ (SM). The grey ribbon around each curve indicates the 95% confidence interval.

**
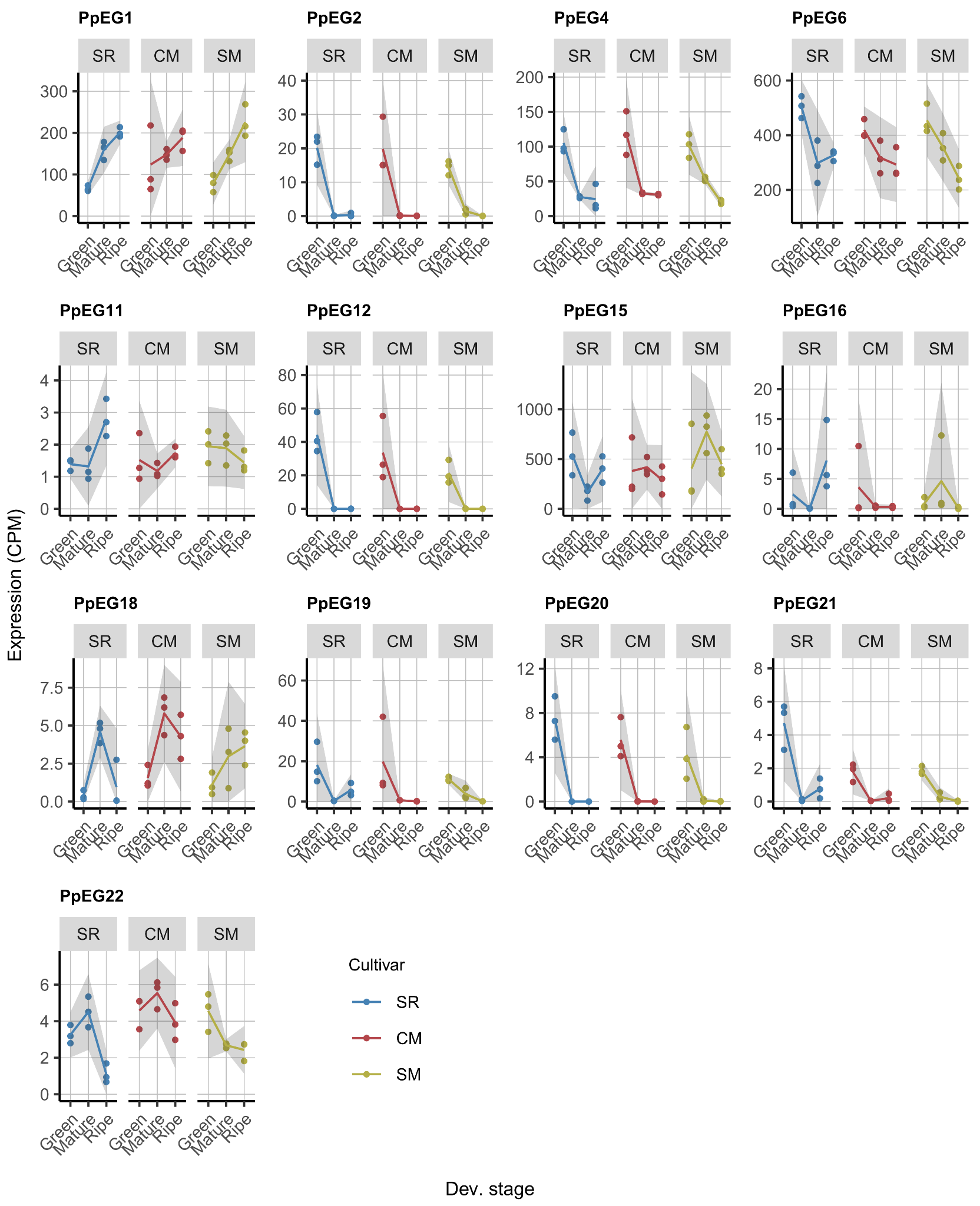
**

**Fig. S13 Expression patterns of cell wall loosening genes, *EG*.** Each facet represents the expression dynamics in flesh across the “Green”, “Mature”, and “Ripe” stages for ‘Santa Rosa’ (SR), ‘Casselman’ (CM), and ‘Sweet Miriam’ (SM). The grey ribbon around each curve indicates the 95% confidence interval.

**
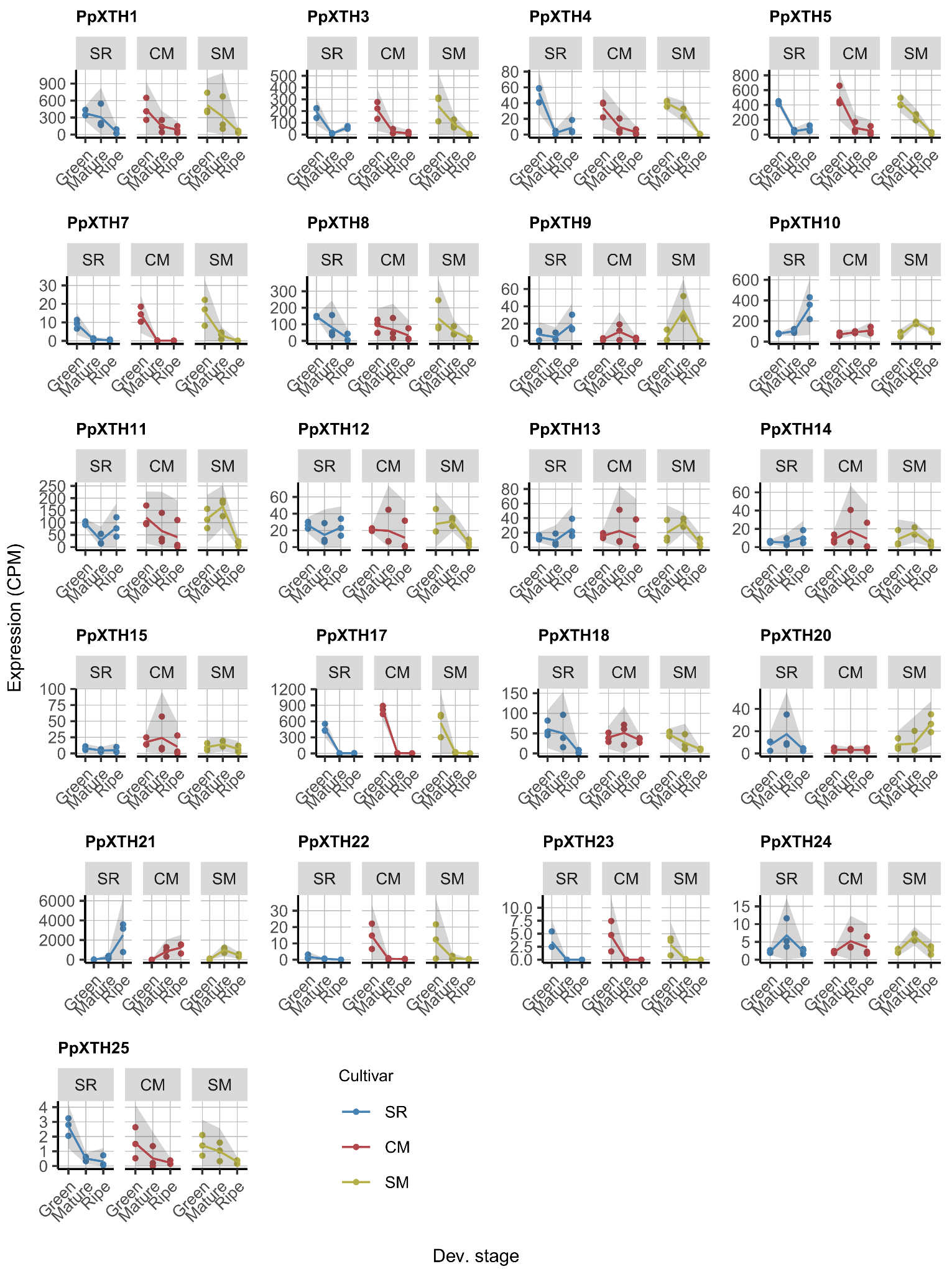
Fig. S14 Expression patterns of cell wall loosening genes, *XTH*.** Each facet represents the expression dynamics in flesh across the “Green”, “Mature”, and “Ripe” stages for ‘Santa Rosa’ (SR), ‘Casselman’ (CM), and ‘Sweet Miriam’ (SM). The grey ribbon around each curve indicates the 95% confidence interval.


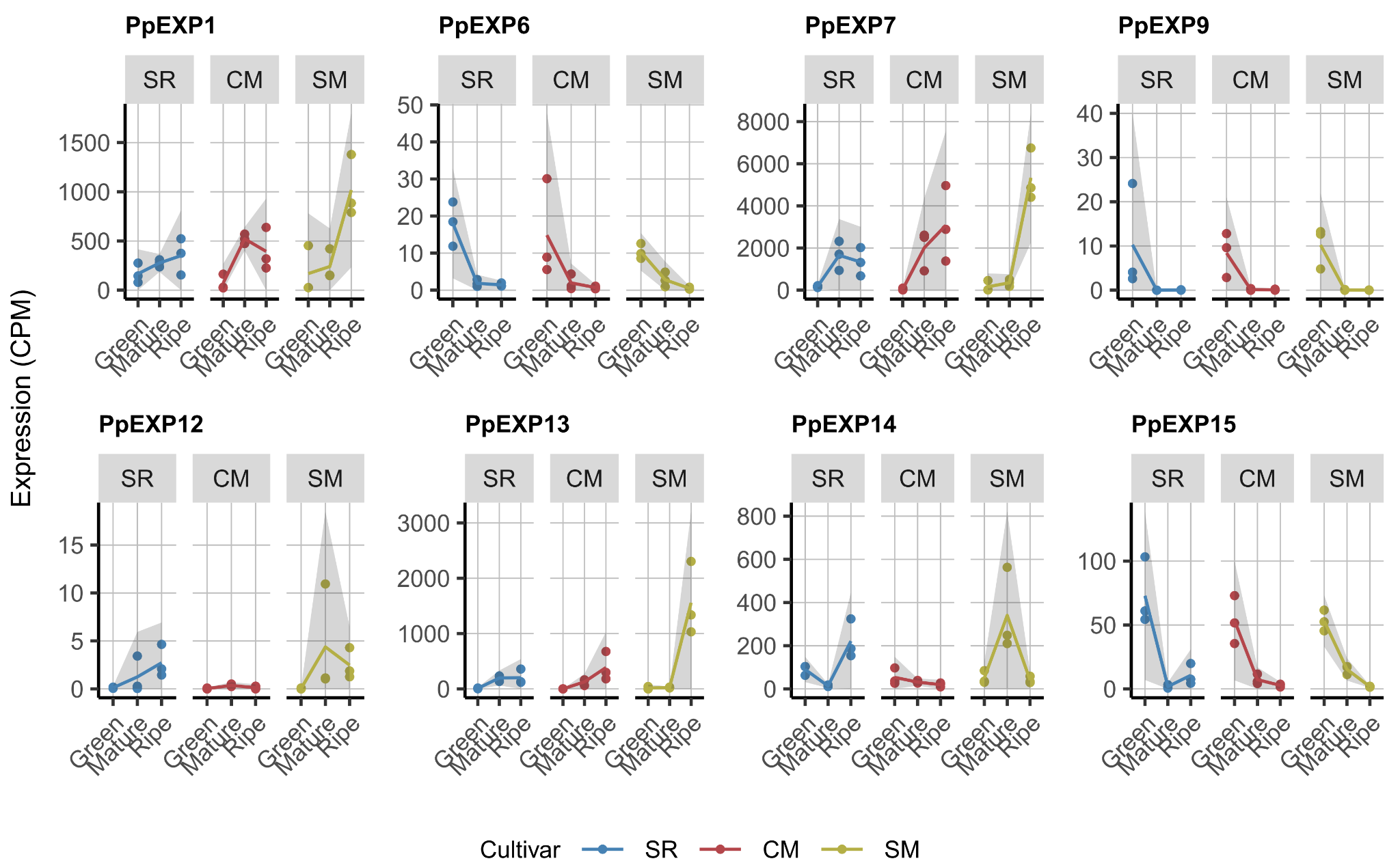


**Fig. S15 Expression patterns of cell wall loosening genes, *EXP*.** Each facet represents the expression dynamics in flesh across the “Green”, “Mature”, and “Ripe” stages for ‘Santa Rosa’ (SR), ‘Casselman’ (CM), and ‘Sweet Miriam’ (SM). The grey ribbon around each curve indicates the 95% confidence interval.


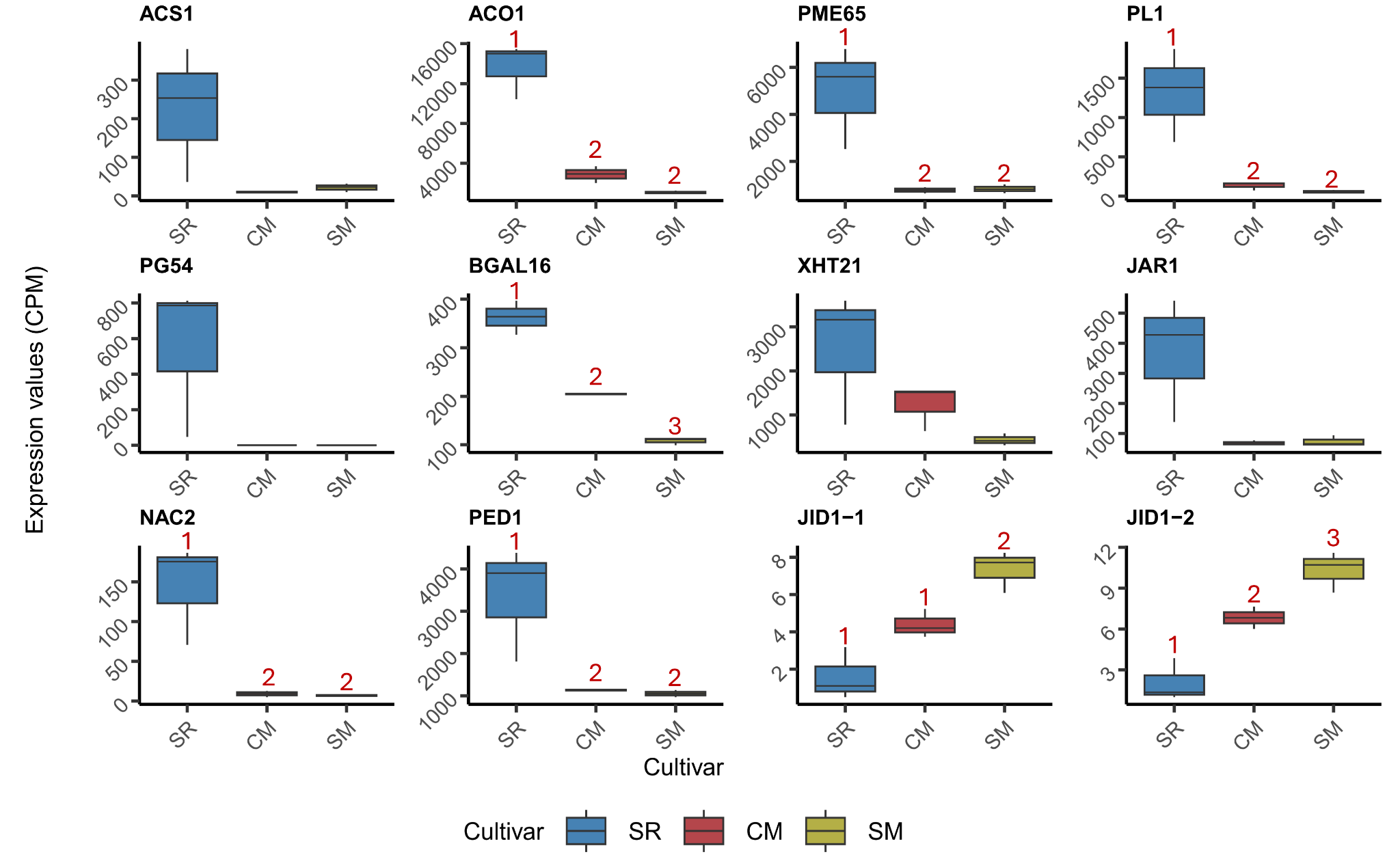


**Fig. S16. Expression differences of target genes among ‘Santa Rosa’ (SR), ‘Casselman’ (CM), and ‘Sweet Miriam’ (SM) at the “Ripe” stage in our RNA-seq data.** This figure extracts target gene expression from Fig. 2 and Fig. S8–S15 at the “Ripe” stage. Boxes, colored by cultivar, represent the interquartile range, with horizontal lines indicating the median. Whiskers extend to the minimum and maximum values within 1.5 times the interquartile range. Numbers above the boxes represent results from Tukey’s method for multiple pairwise comparisons, where different numbers indicate statistically significant differences (*p* < 0.05).


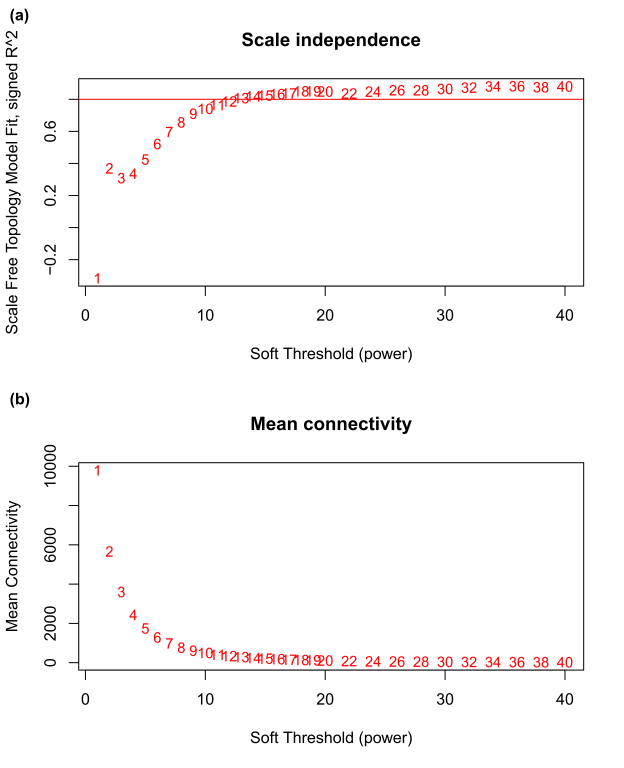


**Fig. S17. Scale independence (a) and mean connectivity (b) for determining the soft-thresholding powers in Weighted gene co-expression network analysis (WGCNA).** The x-axis for both plots represents the soft-thresholding power. The y-axis in (a) represents the scale-free fit index, and in (b) represents the mean connectivity. WGCNA was performed using the WGCNA package (v1.73) with a single-block, signed network, and power = 16.

**Table S1 Summary of plum sample collection and environmental conditions.** This table details the collection of plum samples used in this study, including cultivar, developmental stage, collection date, climacteric type, localization, and temperature conditions. “Month avg temp (°C)” represents the average monthly temperature (in degrees Celsius) at the collection site, obtained from the publicly available Weather Underground database (https://www.wunderground.com/).

| Year | Cultivar | Developmental stage | Date | Climacteric type | Localization | Month avg tmp (°C) |
| --- | --- | --- | --- | --- | --- | --- |
| 2017 | Santa Rosa | Green | 5/30/2017 | Climacteric | Reedley, CA | 24.16 |
| 2017 | Santa Rosa | Mature | 6/21/2017 | Climacteric | Reedley, CA | 33.43 |
| 2017 | Santa Rosa | Ripe | 6/21/2017 | Climacteric | Reedley, CA | 33.43 |
| 2017 | Casselman | Green | 5/30/2017 | Suppressed-  climacteric | Reedley,CA | 24.16 |
| 2017 | Casselman | Mature | 8/8/2017 | Suppressed-  climacteric | Reedley,CA | 28.84 |
| 2017 | Casselman | Ripe | 8/21/2017 | Suppressed-  climacteric | Reedley,CA | 28.29 |
| 2017 | Sweet Miriam | Green | 5/30/2017 | Non-climacteric | Reedley, CA | 24.16 |
| 2017 | Sweet Miriam | Mature | 9/18/2017 | Non-climacteric | Reedley, CA | 20.13 |
| 2017 | Sweet Miriam | Ripe | 10/4/2017 | Non-climacteric | Reedley, CA | 19.03 |
| 2024 | Santa Rosa | Ripe | 7/27/2024 | Climacteric | Brentwood, CA | 24.07 |
| 2024 | Friar | Ripe | 7/29/2024 | Climacteric | Reedley, CA | 28.97 |
| 2024 | Fortune | Ripe | 7/29/2024 | Climacteric | Reedley, CA | 28.97 |
| 2024 | Casselman | Ripe | 8/13/2024 | Suppressed-  climacteric | Reedley, CA | 28.88 |
| 2024 | Late Santa Rosa | Ripe | 8/26/2024 | Suppressed-  climacteric | Vacaville, CA | 23.36 |
| 2024 | Late Santa Rosa | Ripe | 8/26/2024 | Suppressed-  climacteric | Sebastopol, CA | 19.07 |
| 2024 | Angeleno | Ripe | 9/9/2024 | Suppressed-  climacteric | Reedley, CA | 27.05 |
| 2024 | Sweet Miriam | Ripe | 9/9/2024 | Non-climacteric | Reedley, CA | 27.05 |

**Table S2. Comprehensive list of ethylene signaling and softening-related genes across gene families.** EC numbers were obtained using Phytozome. “Family” columns indicate the gene families associated with each protein or enzyme, as predicted by PLAZA Dicot.

**Table S3 qPCR Primers used in this article.**

| Primer Name | Sequence | Gene ID |
| --- | --- | --- |
| IF5A_qF2 | TCCCCATGTCAACCGTACTGACT | Prupe.1G280900 |
| IF5A_qR2 | CCAAAGCCATCCTTGATCTGCGT | Prupe.1G280900 |
| ACS1-qF2 | ACTTCGATGGGTGGAAGGCG | Prupe.2G176900 |
| ACS1-qR2 | TCGATCAAGTCGAAGGAAAGCTGG | Prupe.2G176900 |
| ACO1-qF1 | GGAGAAAGAAGCAGAGGAGAAG | Prupe.3G209900 |
| ACO1-qR1 | GAACTTGAGGCCAGCATAGAG | Prupe.3G209900 |
| PME65_qF1 | TCTGAGCGCGACAAGACTG | Prupe.7G192800 |
| PME65_qR1 | TACTTGACCCGACCCGTTGAG | Prupe.7G192800 |
| PL1_qF1 | GTCTCCATCTTCGGGTCCAGC | Prupe.1G060900 |
| PL1_qR1 | CCCAGCAGCATCACCTCATTGT | Prupe.1G060900 |
| PG54_qF2 | CCAGCGGAGCAACGACTTTGG | Prupe.4G261900 |
| PG54_qR2 | AGCAGAAACCCTGACACCTTGC | Prupe.4G261900 |
| BGAL16_qF1 | CCTTGCTTAGTGCAATGGTCGG | Prupe.1G492800 |
| BGAL16_qR1 | TCTCCATCCAGCCCAACCTGA | Prupe.1G492800 |
| XTH21_qF2 | GGAACCCTACTGAGATCATATTCCT | Prupe.6G110300 |
| XTH21_qR2 | CCTCTGTGGCCCATGTTGAT | Prupe.6G110300 |
| JAR1_qF3 | CATGTGCCGGAGCAATCTTCT | Prupe.2G184100 |
| JAR1_qR3 | CTGTGGACAAGTCTGCATGGC | Prupe.2G184100 |
| NAC2_qF1 | TCGTGTTCTACGCTGGCAAAGC | Prupe.4G279600 |
| NAC2_qR1 | ACAGCACCCAATCATCAAGCCTCAA | Prupe.4G279600 |
| PED1_qF1 | TAGCACCTGTTTTGAAGGCGG | Prupe.1G003300 |
| PED1_qR1 | GCAGCCATCCTGCATTCACT | Prupe.1G003300 |
| JID1_qF1 | TTGGAGATAGAGGAGCAGTGCAG | Prupe.1G035800 and Prupe.1G036000 |
| JID1_qR1 | TAGCAAACCACAACAGAGACAGTT | Prupe.1G035800 and Prupe.1G036000 |

**Table S4. GO enrichment analysis of differentially expressed genes (DEGs) that are upregulated and downregulated during the climacteric stage in SR.** The DGE numbers column indicates the number of DEGs associated with each GO ID. The GO aspects column classifies GO terms into three domains: Molecular Function (MF), Cellular Component (CC), and Biological Process (BP).

**Table S5: Hub genes in the Blue and Brown modules.** The top 10 genes with the highest connectivity were identified as hub genes. Annotations from PLAZA Dicot and NCBI Gene are included.
